# The time and place of European admixture in Ashkenazi Jewish history

**DOI:** 10.1371/journal.pgen.1006644

**Published:** 2017-04-04

**Authors:** James Xue, Todd Lencz, Ariel Darvasi, Itsik Pe’er, Shai Carmi

**Affiliations:** 1 Department of Computer Science, Columbia University, New York, New York, United States of America; 2 Department of Organismic and Evolutionary Biology, Harvard University, Cambridge, Massachusetts, United States of America; 3 Center for Psychiatric Neuroscience, The Feinstein Institute for Medical Research, North Shore-Long Island Jewish Health System, Manhasset, New York, United States of America; 4 Department of Psychiatry, Division of Research, The Zucker Hillside Hospital Division of the North Shore–Long Island Jewish Health System, Glen Oaks, New York, United States of America; 5 Departments of Psychiatry and Molecular Medicine, Hofstra Northwell School of Medicine, Hempstead, New York, United States of America; 6 Department of Genetics, The Alexander Silberman Institute of Life Sciences, The Hebrew University of Jerusalem, Jerusalem, Israel; 7 Department of Systems Biology, Columbia University, New York, New York, United States of America; 8 Braun School of Public Health and Community Medicine, The Hebrew University of Jerusalem, Ein Kerem, Jerusalem, Israel; University of California San Francisco, UNITED STATES

## Abstract

The Ashkenazi Jewish (AJ) population is important in genetics due to its high rate of Mendelian disorders. AJ appeared in Europe in the 10^th^ century, and their ancestry is thought to comprise European (EU) and Middle-Eastern (ME) components. However, both the time and place of admixture are subject to debate. Here, we attempt to characterize the AJ admixture history using a careful application of new and existing methods on a large AJ sample. Our main approach was based on local ancestry inference, in which we first classified each AJ genomic segment as EU or ME, and then compared allele frequencies along the EU segments to those of different EU populations. The contribution of each EU source was also estimated using *GLOBETROTTER* and haplotype sharing. The time of admixture was inferred based on multiple statistics, including ME segment lengths, the total EU ancestry per chromosome, and the correlation of ancestries along the chromosome. The major source of EU ancestry in AJ was found to be Southern Europe (≈60–80% of EU ancestry), with the rest being likely Eastern European. The inferred admixture time was ≈30 generations ago, but multiple lines of evidence suggest that it represents an average over two or more events, pre- and post-dating the founder event experienced by AJ in late medieval times. The time of the pre-bottleneck admixture event, which was likely Southern European, was estimated to ≈25–50 generations ago.

## Author summary

The Ashkenazi Jewish population has resided in Europe for much of its 1000-year existence. However, its ethnic and geographic origins are controversial, due to the scarcity of reliable historical records. Previous genetic studies have found links to Middle-Eastern and European ancestries, but the admixture history has not been studied in detail yet, partly due to technical difficulties in disentangling signals from multiple admixture events. Here, we present an in-depth analysis of the sources of European gene flow and the time of admixture events by using multiple new and existing methods and extensive simulations. Our results suggest a model of at least two events of European admixture. One event slightly pre-dated a late medieval founder event and was likely from a Southern European source. Another event post-dated the founder event and likely occurred in Eastern Europe. These results, as well as the methods introduced, will be highly valuable for geneticists and other researchers interested in Ashkenazi Jewish origins.

## Introduction

Ashkenazi Jews (AJ), numbering approximately 10 million worldwide [1], are individuals of Jewish ancestry with a recent origin in Eastern Europe [2]. The first individuals to identify as Ashkenazi appeared in Northern France and the Rhineland (Germany) around the 10^th^ century [3]. Three centuries later, Ashkenazi communities emerged in Poland, but the source(s) of migration are not completely clear. The Ashkenazi communities in Poland have grown rapidly, reaching, by the 20^th^ century, millions in size and a wide geographic spread across Europe [2].

Due to the relative scarcity of relevant historical records, the ethnic origins of present-day Ashkenazi Jews are debated [2], and in such a setting, genetic data provides crucial information. A number of recent studies have shown that Ashkenazi individuals have genetic ancestry intermediate between European (EU) and Middle-Eastern (ME) sources [4–8], consistent with the long-held theory of a Levantine origin followed by partial assimilation in Europe. The estimated amount of accumulated EU gene flow varied across studies, with the most recent ones, employing genome-wide data, converging to a contribution of around 50% of the AJ ancestry [4, 7, 9].

Despite these advances, little is known about the identity of the European admixing population(s) and the time of the admixture events [2, 10]. Speculations abound, due to the wide geographic dispersion of the Jewish populations since medieval times, but with very few historical records to support any claim [2]. Further complicating the picture is an Ashkenazi-specific founder event that has taken place less than a millennium ago, as manifested by elevated frequencies of disease mutations [11, 12], reduced genetic diversity [13, 14], and an abundance of long tracts of identity-by-descent [9, 15, 16]. Results from our recent study [9] were not decisive regarding the relative times of the European admixture and the founder event, calling for a more in-depth investigation.

A number of previous population genetic studies have attempted, sometimes implicitly, to “localize” the Ashkenazi genomes to a single geographic region or source population [4–6, 17]. However, such approaches may be confounded by the mixed EU and ME Ashkenazi ancestry, which necessarily implies the existence of multiple sources. Here, we overcome this obstacle, following studies in other populations [18, 19], by performing a preliminary step of *local ancestry inference* (LAI), in which each locus in each Ashkenazi genome is assigned as either EU or ME. Following LAI, the source population of the European and Middle-Eastern “sub-genomes” can be independently localized.

We begin our analysis by testing the ability of available LAI software to correctly infer ancestries for simulated EU/ME genomes. Proceeding with *RFMix*, we apply LAI to Ashkenazi SNP array data, and use a maximum likelihood approach to localize, separately, the EU and ME sources. We correct bias introduced by the method using simulations, and show that it is robust to potential errors in LAI. We also employ other methods based on allele frequency divergence between Ashkenazi Jews and other populations, although they turn out to be less informative. To estimate the time of admixture, we first use the lengths of EU and ME tracts and the decay in ancestry correlation along the genome. We further introduce a new method for dating admixture times based the genome-wide EU or ME ancestry proportions. We again remove bias from all methods using simulations. We integrate these results with an analysis of identity-by-descent (IBD) sharing both within AJ and between AJ and other populations. Finally, we compare our estimates to those produced by the *GLOBETROTTER* suite [20–22]. Our results suggest that the European gene flow was predominantly Southern European (≈60–80%), with the remaining contribution either from Western or (more likely) Eastern Europe. The time of admixture, under a model of a single event, was estimated at ≈30 generations ago. However, this admixture time is likely the average of at least two distinct events. We propose that admixture with Southern Europeans pre-dated the late medieval founder event, whereas the admixture event in Eastern Europe was more recent.

## Results

### Data collection

SNP arrays for Ashkenazi Jewish individuals were available from the schizophrenia study reported by Lencz et al., 2013 [23] (see also [24]). SNP arrays for European and Middle-Eastern populations were collected from several sources (Table 1). All genotypes were uniformly cleaned, merged, and phased (Methods), resulting in 2540 AJ, 543 Europeans, and 293 Middle-Easterners genotyped at 252,358 SNPs. Note that while there are additional studies in these populations, we restricted ourselves to (publicly available) Illumina array data to guarantee a sufficient number of remaining SNPs after merging all datasets. We divided the European genomes into four regions: Iberia, North-Western Europe (henceforth Western Europe), Eastern Europe, and Southern Europe (Italy and Greece). The Middle-Eastern genomes were divided into three regions: Levant, Southern Middle-East, and Druze. See Table 1 for further details and S1 Fig for a PCA plot [25] supporting the partition into the indicated regions.

**Table 1:** The populations and datasets used in our analysis.

Region	Sub-region	Populations included	Count	Sources
Ashkenazi			2540	Lencz et al., 2013 [23] (Illumina HumanOmni1-Quad)
Europe	West-EU	Orcadian; French; CEU; GBR	217	Behar et al., 2010 [6] (Illumina 610k, 650k) Behar et al., 2013 [5] (Illumina 610k, 650k, 660k, 730k, 1M) HGDP [26] (Illumina 650k) 1000 Genomes [27] (Illumina Omni 2.5M)
	East-EU	Belarusian; Lithuanian; Ukrainian; Polish; Russian	112	
	South-EU	Italians: Tuscan, Abruzzo, Sicilian, Bergamo; Greek	162	
	Iberia		52	
Middle-East	Levant	Palestinian; Lebanese; Jordanian; Syrian	146	Behar et al., 2010 [6] (Illumina 610k, 650k) Behar et al., 2013 [5] (Illumina 610k, 650k, 660k, 730k, 1M) HGDP [26] (Illumina 650k) Haber et al., 2013 [28] (Illumina 610k, 660k)
	South-ME	Egyptian; Bedouin; Saudi	77	
	Druze	Israeli and Lebanese	70	

### Inferring the place of admixture using local ancestry inference

#### Calibration of the local ancestry inference method

In local ancestry inference (LAI), each region of the genome of each admixed individual is assigned an ancestry from one the reference panels. After evaluating the performance of a number of LAI tools on admixture between closely related populations (S1 Text section 1), we selected *RFMix* [29], which is based on a random forest classifier for each genomic window followed by smoothing by a conditional random field. When running *RFMix*, we did not iterate over the inference process using the already classified individuals (the Expectation-Maximization step), as we found that accuracy did not improve (Methods) and we wanted to avoid bias due to the widespread haplotype sharing in AJ. We also did not filter SNPs by the quality of their local ancestry assignment, as we found that such filtering substantially biases downstream inferences (S1 Text section 1). Finally, we downsampled the reference panels to balance the sizes of the European and Middle-Eastern groups, as well as balance the number of genomes from each European region (Methods).

Running *RFMix* on the AJ genomes with our EU and ME reference panels and summing up the lengths of all tracts assigned to each ancestry, the genome-wide ancestry was ≈53% EU and ≈47% ME, consistent with our previous estimate based on a smaller sequencing panel [9]. Our simulations suggested that the accuracy of LAI for an EU-ME admixed population is only around ≈70%, much lower than the near-perfect accuracy observed for cross-continental admixture (e.g., [29–33]). The local ancestry assignment is nevertheless non-random, and therefore, with proper accounting for errors (below), can be informative on the place and time of admixture events.

#### Geographic localization of the EU component of the AJ genomes

Following the deconvolution of segments of EU and ME ancestry, we focused on the regional ancestry of the European segments. We initially followed refs. [18, 19] and attempted to apply *PCAMask* to the EU subset of the AJ genomes. However, *PCAMask*’s results were inconsistent across runs and parameter values (see S1 Text section 2 and [34]). We therefore developed a simple naïve Bayes approach. We first thinned the SNPs to assure linkage equilibrium between the remaining SNPs. We then computed the allele frequencies of the SNPs in the four EU sub-regions: Southern EU, Western EU, Eastern EU, and Iberia. Then, for each haploid chromosome, we computed the log-likelihood of the European assigned part of the chromosome to come from each of the four regions, as a product of its allele frequencies. The inferred source of each chromosome was the EU region with the maximum likelihood for that chromosome.

**Fig 1:**
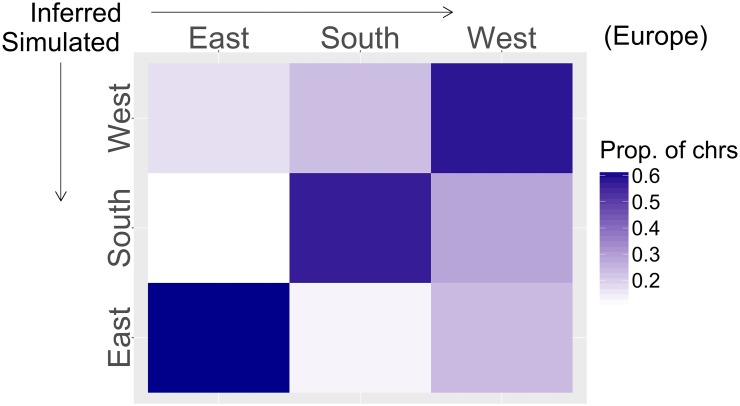
Simulation results for our localization pipeline. In each row, admixed genomes were simulated with sources from the Levant (50%) and one European region (50%). Columns correspond to the inferred proportion of the chromosomes classified as each potential source. The source of each chromosome was chosen as the one that maximizes the likelihood of observing the alleles designated by *RFMix* as European.

Initial inspection of the results revealed that Iberia had consistently lower likelihood than the other regions. Since the Iberian panel was the smallest and sample sizes had to be balanced across regions, we removed the Iberian genomes from the reference panel, thereby increasing the sample size for the other regions (Methods). To determine whether the true ancestry could theoretically be recovered given a single European source, we generated simulated chromosomes using genomes not included in the *RFMix* reference panel. Each simulated chromosome was a mosaic of segments from Middle-Eastern and European genomes, and segment lengths were exponentially distributed, according to the expected parameters of a symmetric admixture event occurring 30 generations ago (Methods). In each simulation experiment, the identity of the European source region was varied, and the proportion of chromosomes inferred to have each EU region as their source was calculated. We found that the true EU source region had the highest proportion of classified chromosomes in all cases (Fig 1). This result indicates that localization of the European source is feasible, despite the noise and bias in local ancestry inference between closely related populations such as Middle-Easterners and Europeans.

For AJ, we found that Southern Europe was the most likely EU source for the largest proportion of the AJ chromosomes. Specifically, 43.2% of the AJ chromosomes had Southern EU as their most likely source, 35.4% had Western EU, and 18.8% had Eastern EU (the proportions do not precisely sum to 1, as we also allowed chromosomes to be classified as Middle-Eastern). These results imply that Southern Europe was the dominant source of European gene flow into AJ.

We observed that in simulations of admixed genomes, the Middle-Eastern regional source could have also been recovered by running the same localization pipeline. Applying that pipeline to the AJ genomes, we identified Levant as the most likely ME source: the proportions of chromosomes classified as Levantine was 51.6%, compared to 21.7% and 22.2% classified as Druze and Southern ME, respectively.

While these results indicate a sizeable contribution of ancestry from Southern Europe and the Levant, we stress that these quantities do not directly correspond to the proportion of ancestry contributed by each source. We attempt to infer those proportions in the next section.

#### Inferring the proportion of ancestry contributed by each EU and ME region

**Fig 2:**
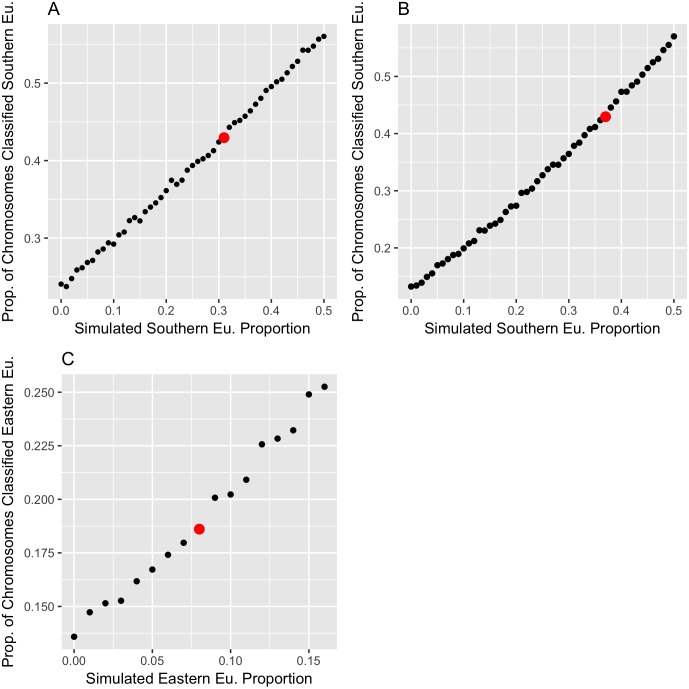
Inference of the proportion of Ashkenazi ancestry derived from each European region. We simulated admixed chromosomes with European and Middle-Eastern ancestries, where the ME ancestry was fixed to the Levant region and to 50% of the overall ancestry. We then varied the sources of the remaining European ancestry to determine which ancestry proportions most closely match the AJ data. In (A), the simulated EU components were Southern and Western EU. For each given proportion of Southern EU ancestry, we used our LAI-based pipeline to compute the proportion of chromosomes classified as Southern European. The best match to the proportion of thus classified chromosomes observed in the real AJ data (red dot) was found when the true simulated Southern EU ancestry was 31% of the total. In (B), the same simulation procedure was repeated, except that the simulated EU components were Southern and Eastern EU. The inferred proportion of Southern EU ancestry in AJ was 37%. (C) We fixed the Southern EU contribution to 34%, the average of its estimates from (A) and (B), and varied the remaining 16% between Western and Eastern EU. The simulations suggest that the closest match to the real results is at roughly equal contribution (8%) from Western and Eastern EU.

To quantitatively estimate the contribution of each subcontinental European region, we used the above-mentioned proportions of chromosomes classified to each EU region as summary statistics, and matched them to simulations in which the proportions of ancestry contributed by each region is known. Specifically, we performed 4-way admixture simulations between individuals of Levantine, Southern European, Eastern European, and Western European origin. In these simulations, we fixed the Levantine admixture proportion to 50% and varied the proportions of the different European regions. We then used a grid search to find the ancestry proportions that best fit the observed fraction of AJ chromosomes classified as each ancestry. The simulation results (Fig 2) suggest that the European component of the AJ cohort is 34% Southern EU, 8% Western EU, and 8% Eastern EU. This analysis thus suggests that roughly 70% of EU ancestry in AJ is Southern European. Using bootstrapping (S1 Text section 3), the 95% confidence interval of the Southern EU ancestry was [33,35]% and that of Eastern EU was [8,9]%. However, bootstrapping does not account for any systematic biases, which in this case are of larger magnitude (S1 Text section 3 and below).

To estimate the magnitude of the minor ME components, we repeated a procedure similar to that used for the European component. Specifically, we simulated admixed genomes in which the European ancestries were fixed to the proportions inferred above (34% Southern EU, 8% Western EU, and 8% Eastern EU), and varied the proportion of Levant vs Druze ancestry and then Levant vs Southern ME ancestry. The best match to the AJ data was obtained (in both cases) when the Levant ancestry was almost entirely exclusive (45% out of the total 50% ME ancestry; the magnitude of the minor components was close to zero also when we simulated 50% Southern EU ancestry). This result supports a predominantly Levantine origin for the ME ancestry in AJ, and justifies using the Levantine genomes for the ME ancestry in our simulations.

In S1 Text section 3, we describe simulations that demonstrate the robustness of our pipeline to changing the proportion of simulated Levantine ancestry, including Iberia in the reference panel, and excluding from the panel the true Middle-Eastern and/or European ancestral sources.

### Inferring the time of admixture using local ancestry inference

#### Mean segment length

**Fig 3:**
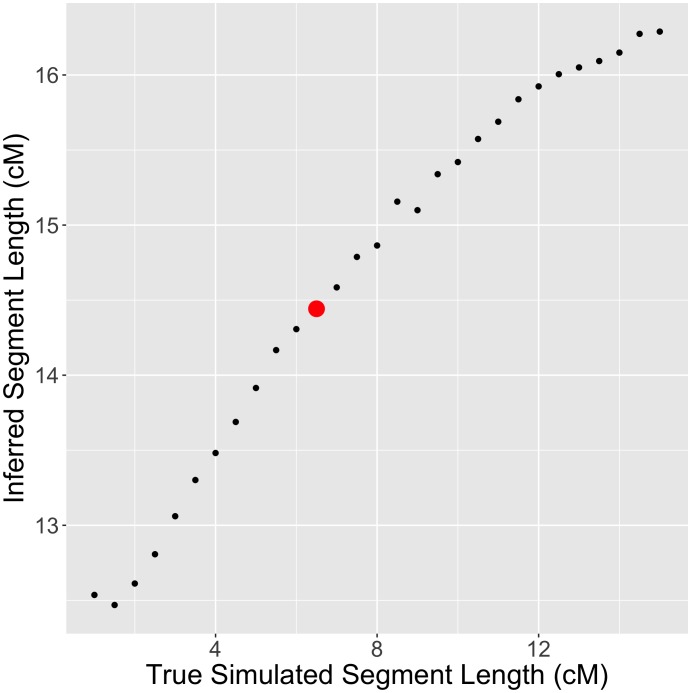
Inferring the AJ admixture time using the lengths of admixture segments. The mean length of *RFMix*-inferred Middle-Eastern segments is plotted vs the mean simulated length, which is inversely related to the simulated admixture time. The red dot corresponds to the observed mean segment length in the real AJ data.

Consider a model of a “pulse” admixture between two populations, *t* generations ago, where the first population has contributed a fraction *q* of the ancestry. The mean length (in Morgans) of segments coming from the second source is 1/(*qt*) [35]. In the case of AJ, where the source populations are EU and ME, we estimated *q* above (EU ancestry fraction) to be ≈53%. Therefore, the mean ME segment length is expected to be informative on the time of admixture *t*. The mean ME segment length was ≈14cM; however, we noticed that in simulations, the *RFMix*-inferred segment lengths were significantly overestimated. To correct for that, we used simulations to find the admixture time that yielded *RFMix*-inferred segment lengths that best matched the real AJ data. We fixed the ancestry proportions to the ones inferred above for AJ (50% ME, 34% Southern EU, 8% Western EU, and 8% Eastern EU), and varied the admixture time. We then plotted the *RFMix*-inferred ME segment length vs the simulated segment lengths (Fig 3). The simulated mean segment length that corresponded to the observed AJ value was around 6.6cM, implying an admixture time of ≈29 generations ago (bootstrapping 95% confidence interval: [27,30] generations).

#### Chromosome-wide ancestry proportions

Beyond mean segment lengths, the proportion of ancestry per chromosome that descends from each ancestral population is also informative on the time of admixture [36, 37], since the longer the time after admixture, the smaller its variance [35]. While ancestry proportions contain less information than segment lengths, they are potentially more robust to misidentification of the segments boundaries. Building on models from refs. [35, 38, 39], we derived a new analytical expression for the distribution of ancestry proportions (for either phased or unphased data) given the initial admixture proportions and admixture time (Methods). This led to a maximum likelihood estimator of the admixture time and the initial proportions. For admixture between highly diverged populations, the method is expected to work well for intermediate admixture times (e.g., 10<t<100 generations [40]), as we demonstrated using simulations in which the true segment boundaries were known (S2 Fig).

**Fig 4:**
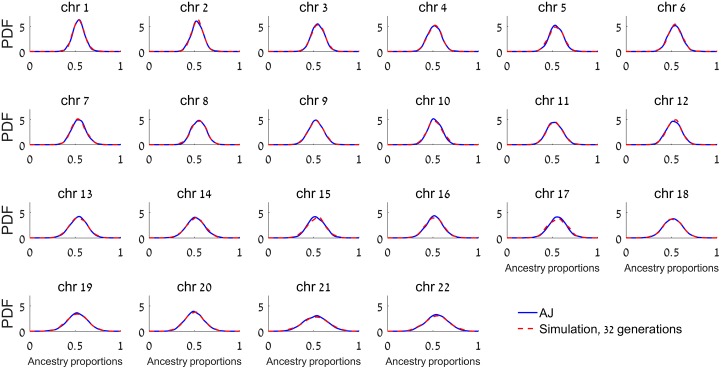
The Probability Density Function (PDF) of ancestry proportions in AJ and in simulations. The ancestry proportions in AJ were computed using LAI (*RFMix*). Simulations are of 1000 genomes with a history of an admixture pulse 32 generations ago between Middle-Eastern, Southern EU, Eastern EU, and Western EU populations. The density was estimated using a normal kernel. The admixture time was estimated by fitting the average standard deviation of the ancestry proportions across all chromosomes to the AJ data, where each chromosome was weighted by the square root of its length in cM. The confidence interval ([31,37] generations) was obtained by resampling AJ individuals, with replacement, 1000 times.

To apply our method to AJ, we used the LAI results and summed up the lengths of European and Middle-Eastern segments. However, our simulations showed that for Southern EU/ME admixture, the correlation between true and inferred ancestry proportions is only *r*^*2*^ ≈ 0.11 (S3 Fig), and therefore, we could not directly apply our method. To correct for the distortion of the distribution due to local ancestry inference, we again used EU/ME admixture simulations, and matched the variance of the AJ distribution to that of genomes simulated under admixture times between 10 to 60 generations. We found that the best fit to the AJ data, given a 4-way admixture model (Middle-Eastern, Southern EU, Eastern EU, and Western EU with proportions 50:34:8:8 (%), respectively) was obtained with admixture time of 32 generations (Fig 4) (95% bootstrapping confidence interval [31,37] generations), close to the time inferred above using the mean segment lengths.

#### The number of admixture events

In light of identifying multiple EU ancestral sources, the assumption of pulse admixture might be unrealistic. In S1 Text section 6, we analytically derive the distribution of segment lengths and ancestry proportions for a double admixture model, where the initial admixture event was followed by a second contribution from one of the sources. However, we observed that the ancestry proportions from this model can sometimes be fitted well by pulse admixture. Given this and the considerable noise introduced by LAI, directly estimating the parameters of multiple admixture events is unlikely to be reliable.

To overcome this problem, we first note that the inferred single admixture time still imposes some constraints on the admixture times and proportions in a double admixture model (Methods). Additionally, we notice that the estimated admixture time (≈30–35 generations) is very close to the time estimated for the AJ bottleneck event [9, 16]. If indeed two distinct admixture events have occurred, the single estimated admixture time represents a weighted average of the times of the two events (Methods). For that weighted average to coincide with the AJ bottleneck, it is reasonable to assume that one event has pre-dated the bottleneck, while the other has post-dated it, or at least that the two events have occurred at different stages of the bottleneck. This is expected to leave different traces when examining the ancestry of genomic segments with origin at around the time of the bottleneck, compared to the rest of the genome. We apply these insights in the following section.

### The ancestry of Identical-By-Descent (IBD) segments

A number of recent studies have shown that sharing of identical-by-descent (IBD) segments is abundant in the AJ population, and is likely due to a severe bottleneck around 30 generations ago [4, 7, 9, 15, 16]. An open question is the relative timing of the bottleneck and the European gene flow, with our current and past [9] point estimates dating admixture at around or slightly earlier than the bottleneck. Given that most IBD segments but the very long ones (e.g., of length >7cM) coalesce around the time of the bottleneck, we contrast three hypotheses. If admixture completely predated the bottleneck, then IBD segments should have the same EU/ME ancestry proportions as observed genome-wide. If European admixture completely post-dated the bottleneck, then IBD segments should show exclusive ME ancestry. If, on the other hand, European gene flow occurred both before and after the bottleneck, then IBD segments should show an elevated (though not exclusive) ME ancestry compared to the rest of the genome [41–43]. Further, IBD segments of different lengths shared between AJ and other populations could shed light on the geographic origin of each admixture event.

We detected long (>3cM) IBD segments using *Germline* [44] and *Haploscore* [45] (Methods). For segments shared within AJ individuals, we then computed the total amount of genetic material in IBD segments associated with each pair of diploid ancestries, namely, the fraction of SNPs in IBD segments where each of the two individuals sharing the segment has either homozygous EU ancestry, homozygous ME ancestry, or heterozygous ancestry. Clearly, errors in IBD segment detection and local ancestry inference could severely bias the conclusions of such an analysis. Fortunately, we could naturally account for these errors using the observed amount of genetic material in IBD segments shared between individuals labeled homozygous ME and homozygous EU, since the proportion of such segments is a direct measure of the noise level (Methods and S1 Text section 4).

Our results demonstrate an over-representation of Middle-Eastern IBD segments, consistent with two waves of gene flow. Specifically, we estimated the European fraction of the AJ ancestry at the bottleneck as 42%, less than the 53% observed genome-wide (Methods). The contribution of *post*-*bottleneck* European gene flow required to explain these figures is 19% of the AJ ancestry (Methods). Considering only segments of length between [3,7]cM (as longer segments may descend from ancestors even more recent than the bottleneck) slightly increased the inferred magnitude of post-bottleneck gene flow to 22%, or 23% when considering only segments between [3,4]cM.

Given a history of multiple admixture events, a natural question is the geographic source of each event. According to the documented AJ migration history, we speculated that the Southern-European gene flow was pre-bottleneck and that the Western/Eastern European contribution came later. Indeed, we note that the estimated proportion of ≈20% post-bottleneck replacement is close to our above estimate of ≈16% EU gene flow from sources other than Southern-EU as well as to *TreeMix*’s and *Globetrotter*’s results below (and perhaps also with our previous estimate of ≈15% EU ancestry based on AJ and Western European (CEU) data alone [46]). To test this hypothesis, we considered the European ancestry of IBD segments longer than 15cM, which are highly unlikely to predate the bottleneck. The proportion of AJ chromosomes with all regions masked but the >15cM IBD segments inferred by our geographic localization pipeline to be most likely Southern European decreased by 14.8% points compared to the genome-wide results. In contrast, the proportion of AJ chromosomes inferred to be most likely Eastern and Western European increased by 10.2 and 4.5% points, respectively. As a control, when we considered AJ individuals reduced to IBD segments of *any* length, there was no noticeable change.

**Fig 5:**
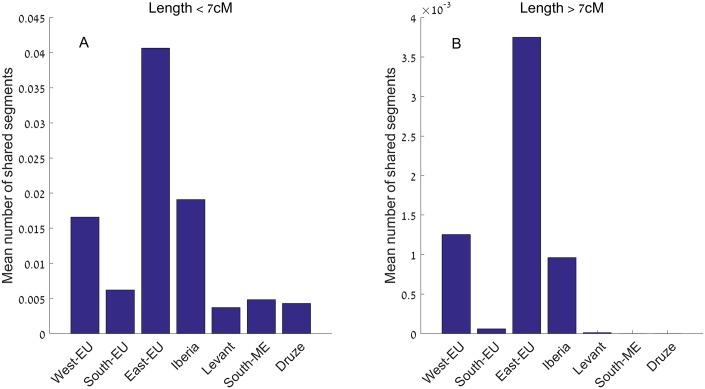
The number of IBD segments shared between Ashkenazi Jews (AJ) and other groups of populations. IBD segments were detected by *Germline* and *Haploscore*, as explained in Methods. The population groups are as in Table 1. Note the different scale of panels (A) and (B) (segments of length between [3,7]cM and >7cM, respectively), and that sharing between AJ and either Southern Europeans or Middle Easterners completely vanishes for the longer (more recent) segments, indicating a relatively older divergence/gene flow. Also note that while sharing with Eastern Europeans is high compared to other groups, it is nevertheless a relatively rare event (≈0.04 segments per pair of individuals), in particular compared to sharing within AJ (≈3.4 segments per pair).

We also considered IBD segments shared *between* AJ and other populations (Fig 5), and observed that the number of segments shared between AJ and Eastern Europeans was ≈6-fold higher than shared between AJ and Southern Europeans (consistent with [5]), with this ratio increasing to ≈60-fold for segments of more recent origin (length >7cM). Further, the number of segments shared with Eastern Europeans was ≈2-fold higher than with Western Europeans or the people of Iberia (P = 5∙10^−3^ for the difference, using permutations of the EU regional labels), pointing to Eastern Europe as the predominant source of *recent* gene flow.

### Inferring the time and source of gene flow using additional methods

#### Decay of admixture linkage disequilibrium (*Alder*), *f*_4_ statistics, and tree structure (*Treemix*)

Refs. [47–49] have shown that linkage disequilibrium (LD) in an admixed chromosome, weighted properly, decays exponentially with genetic distance, and the *Alder* package was implemented to infer the admixture time and the ancestral sources. The admixture time inferred by *Alder* for AJ is broadly consistent with the LAI-based results, at 30–40 generations ago (Table 2; the P-value for admixture was significant under all tests).

**Table 2:** Inferring the AJ admixture time and sources using *Alder*. Admixture times are in generations. The parameters were inferred, for each European region, using *Alder*’s self-determined minimal distance cutoff (rightmost column), above which the admixture LD decay is fitted. The other reference panel was always Middle-Eastern.

	Admixture time	Amplitude	Z-score	Cutoff
Southern Europe	39.8	2.8∙10^−6^	15.2	1.4cM
Eastern Europe	29.6	8.6∙10^−6^	18.1	1.9cM
Western Europe	35.3	8.2∙10^−6^	26.6	1.5cM

For a simple admixture history, the LD curve amplitude increases as the reference population becomes closer to the true ancestral source. The *Alder* results (Table 2) would thus suggest that Eastern and Western Europeans are closer to the source of European gene flow into AJ, in contrast to the LAI-based results. However, when we ran *Alder* on simulated genomes with an admixture event, 30 generations ago, between Levant and Southern/Eastern/Western EU with respective ancestry proportions 50:35:12:3(%), the amplitudes were nearly identical to those of the real data, with the admixture times maintaining the same relative order and slightly overestimated at 34–41 generations ago. In fact, even simulations of pure Levant/Southern EU admixture resulted in higher Western/Eastern EU amplitudes than Southern EU. We thus conclude that, perhaps due to the complex admixture history in Southern Europe, *Alder* cannot infer the true ancestral sources, and that the results are still consistent with a model of predominantly Southern European contribution.

A similar situation was observed when inferring the ancestral tree topology using *f*_*4*_ statistics [48] (S4 and S5 Figs) and *TreeMix* [50] (S6 Fig), both of which rely on the covariance of allele frequencies (or frequency differences) across populations. We measured the *f*_*4*_ statistic for the configuration (X,YRI;AJ,ME), where we used Yoruba (YRI) as an outgroup, and substituted different European regions for X (S4 Fig, part A). The European region that gave the highest value of *f*_*4*_, Eastern Europe (closely matched by Western Europe), is theoretically the one closest to the source of European gene flow. However, simulations with a dominant (and even exclusive) Southern European source resulted in highest *f*_4_ values for Eastern Europe as well. [This discrepancy might be explained, at least partly, by a strong Middle-East to Southern EU migration event [51] (S5 Fig), or by the small component of African ancestry in Southern Europeans [49].] Therefore, these results are still consistent with a dominant Southern EU source for AJ. We used the *f*_*4*_ statistics to infer the fraction of European ancestry in AJ, as explained in Patterson et al. [48]. Assuming that the true source is Southern Europe, the EU ancestry proportion is theoretically given by *f*_*4*_(West-EU,YRI;AJ,ME)/*f*_*4*_(West-EU,YRI;South-EU,ME)≈67% (S4 Fig, part B). However, when simulating genomes with 50% European ancestry, the *f*_4_-inferred fraction came out as 63%; thus, an inferred European ancestry proportion of 67% is broadly consistent with the *RFMix*-based estimate of ≈53%.

We next ran *TreeMix* on AJ, Middle-East, the four European regions (West/East/South/Iberia), and YRI as an outgroup. The inferred tree (S6 Fig) suggests that AJ split first, followed by Middle-Easterners and Europeans. *TreeMix* then predicted replacement of ≈42% of the Southern EU ancestry by Middle-Eastern migration and ≈17% of the AJ ancestry by Eastern European migration, with the only other significant migration events coming from YRI and having much lower magnitude. However again, simulations with a predominantly Southern European ancestry yielded nearly identical results (S6 Fig). Interestingly, in simulations, *TreeMix* correctly estimated ≈13–14% Eastern EU ancestry in AJ when the true value was 12%, and almost no Eastern EU ancestry (≈2%) when none was simulated alongside Southern EU and ME ancestry; however, Eastern EU ancestry was erroneously estimated when the true simulated ancestry alongside Southern EU and ME was Western EU (16%).

To summarize this section, we demonstrated that the raw results returned by *Alder*, *f*-statistics, and *TreeMix* must only be interpreted in light of simulations. Using simulations, the results were overall consistent with our model of an admixture event ≈30–35 generations ago in Southern Europe, with minor contributions of either Western or Eastern Europe.

#### *GLOBETROTTER* analysis

Finally, we considered *GLOBETROTTER* [21], which can infer both the contribution of each ancestral source and the admixture time. The first step in a *GLOBETROTTER* analysis is running *CHROMOPAINTER* [20], in order to determine the proportion of ancestry of each individual that is “copied” from each other individual in the dataset. Then, an *ancestry profile* for each population is reconstructed, representing the contribution of each other population to its ancestry [21, 22]. The inferred ancestry profile for AJ was 5% Western EU, 10% Eastern EU, 30% Levant, and 55% Southern EU. The combined Western and Eastern EU component is in line with our other estimates, as well as the dominance of the Southern EU component. However, the overall European ancestry, ≈70% (or ≈67% after calibration by simulations; S1 Text section 5), is about 15% higher than the LAI-based estimate, as well as our previous results based on whole-genome sequencing [9]. Our detailed simulations (S1 Text section 5) demonstrate that evidence exists to support either estimate. Possibly, the true fraction of EU ancestry is midway around ≈60%.

Using the ancestry profiles calculated in the first step, G*LOBETROTTER* is also able to infer the admixture time and proportions, by assuming that the source groups could themselves be mixtures of the populations in the sample. A single admixture event was inferred for AJ (S1 Text section 5), where the first source, comprising 36% of the total ancestry, was 46% Western EU and 53% Eastern EU. The second source (64% of the total ancestry) was 35% Southern EU and 65% Levant, and the inferred admixture date was 34 generations ago. Our simulations (S1 Text section 5) show that the inferred genome-wide proportion of ≈22% Southern EU ancestry was significantly underestimated (by ≈20%-points) but that the overall inferred EU ancestry (here ≈58%) was accurate. The inferred admixture time was overestimated by ≈10 generations, implying an AJ admixture time 24 generations ago. With these adjustments, the results are broadly consistent with our conclusions so far. However, it remains open to explain the discrepancy between the inferred proportions from the ancestry profiles and the inferred proportions when running the full *GLOBETROTTER* pipeline.

### Bounding possible historical models

We have so far provided multiple estimates for the ancestry proportions from each source and the time of admixture events. We now attempt to bring these estimates together into a single model and provide bounds on the model’s parameters. The results of all analyses (at least once examined in the light of simulations) point to Southern Europe as the European source with the largest contribution. At the same time, relatively large contributions from Western and/or Eastern Europe were also detected, with some analyses (IBD within AJ and between AJ and other sources, and *GLOBETROTTER*) showing stronger support for an Eastern European source. Based on historical plausibility, these admixture events must have happened at different times, implying multiple events. The inferred admixture time, when modeled as a single event, was between 24–37 generations ago across the methods we examined (corrected mean segment length and ancestry proportions, *Alder*, and *GLOBETROTTER*), very close to the time of the AJ bottleneck, previously estimated to ≈25–35 generations ago [9, 16]. Therefore, it is plausible to argue that one admixture event occurred before or early during the bottleneck, while the other event happened after the bottleneck, with the IBD analysis suggesting that the more recent admixture was with Eastern Europeans.

**Fig 6:**
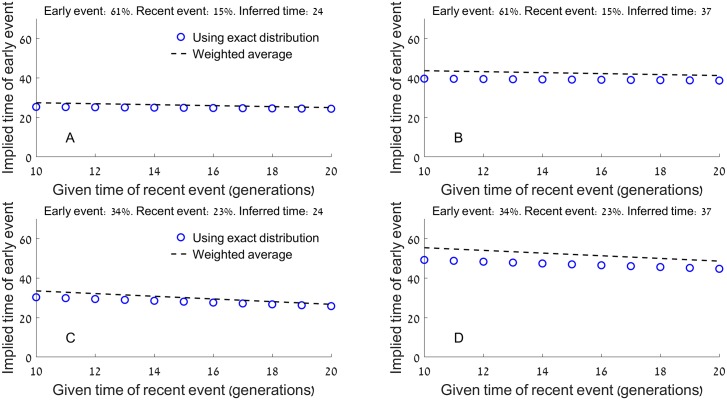
The relationship between the two admixture times in the Ashkenazi history, given bounds on the other admixture parameters. In the model, two populations (**A** and **B**) mixed *t*_*1*_ generations ago (early event; the proportion of ancestry contributed by population **A**, *q*, is indicated in the title of each panel). At a more recent time, *t*_*2*_ generations ago (recent event), migrants from **A** replaced another proportion *μ* of the admixed population (also in the titles). In each panel, we assumed that *q* and *μ* are known, as is the admixture time inferred under the assumption of a pulse admixture model (titles). Under these assumptions, and using Eq (5) in Methods, we plotted the time of the early event (*t*_*1*_) vs the time of the recent event (*t*_*2*_; blue circles). The weighted average (dashed lines) is a simple approximation, in which the time inferred under the pulse model is an average of *t*_*1*_ and *t*_*2*_, weighted by the admixture proportions *q* and *μ*, respectively. In the context of the Ashkenazi Jewish admixture history, population **A** is European and **B** is Middle-Eastern. Panels (A)-(D) represent the bounds on (i) the admixture time inferred under a pulse model (24–37 generations ago); (ii) the admixture proportions at the early and recent events (34–61% and 15–23%, respectively); and (iii) the time of the recent admixture event (10–20 generations ago). These bounds are justified in the main text. The results demonstrate that (i) the weighted average is a reasonable approximation, though the pulse admixture time is influenced more by the early event, perhaps as it results in more admixture tracts; and (ii) the most extreme values of the early AJ event are 24 and 49 generations ago. The lower bound corresponds to the lowest value of the inferred (single event) admixture time, the highest value of the time of the recent admixture event, and the largest contribution of the early event to the overall admixture proportions, and vice versa for the upper bound.

**Fig 7:**
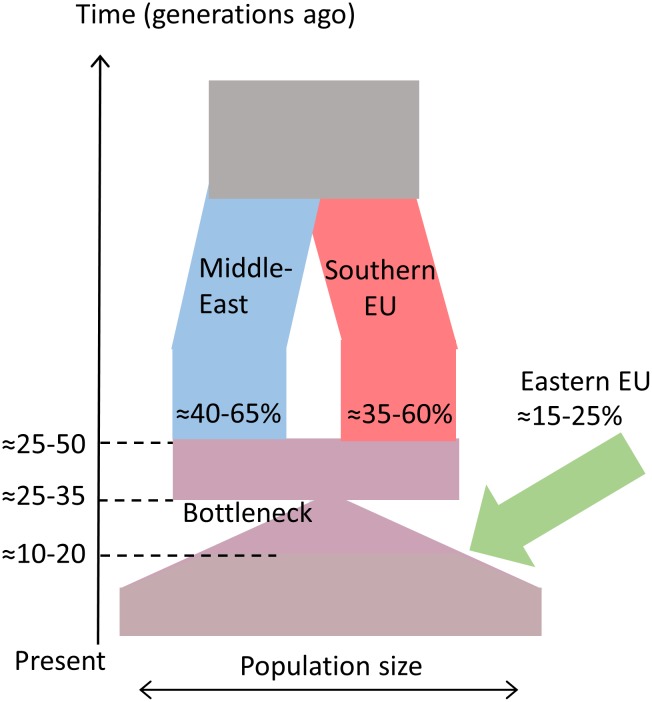
A proposed model for the recent AJ history. The proposed intervals for the dates and admixture proportions are based on multiple methods, as described in the main text.

Based on these arguments, we propose that a minimal model for the AJ admixture history should include substantial pre-bottleneck admixture with Southern Europeans, followed by post-bottleneck admixture on a smaller scale with Western or (more likely) Eastern Europeans. The estimates for the total European ancestry in AJ range from ≈49% using our previous whole-genome sequencing analysis [9], to ≈53% using the LAI analysis here, and ≈67% using the calibrated *Globetrotter* analysis. The proportion of Western/Eastern European ancestry was estimated between ≈15% (*Globetrotter* and the LAI-based localization method), and, if identified as the source of the post-bottleneck admixture, 23% (the IBD analysis). Therefore, the proportion of the Southern European (presumably pre-bottleneck) ancestry in AJ is between ≈26% to ≈52%, corresponding to [34,61]% ancestry at the time of the early admixture. Given these bounds, along with the admixture time estimate based on a single event (24–37 generations ago), we derived a constraint on the admixture times of the pre- and post-bottleneck events (Methods). We further assumed that post-bottleneck admixture happened at most 20 generations ago, when the effective population size has already recovered from the bottleneck (since our estimate of the post-bottleneck admixture proportions relied on the part of the genome *not* shared IBD; see the IBD analysis above and Methods). Finally, we assumed that post-bottleneck admixture happened no more recently than 10 generations ago, since no mass admixture events are known in the past 2–3 centuries of AJ history [52]. The results (Fig 6) show that given these constraints, the pre-bottleneck admixture time is between 24–49 generations ago. Our proposed model is shown in Fig 7.

## Discussion

### Summary and lessons

The ethnic origins of Ashkenazi Jews have fascinated researchers for over a century [53, 54]. The availability of dense genotypes for hundreds of AJ individuals, along with the development of new analysis tools, demonstrated genetic relatedness between AJ and other Jewish groups, and suggested Europe and the Middle-East as putative ancestral sources [4–8, 24]. Here we attempted, for the first time, to create a detailed portrait of the admixture events experienced by AJ during their dwelling in Europe. To this end, we used previously generated genome-wide array data for AJ, European, and Middle-Eastern populations (Table 1), as well as a variety of current and newly developed population genetics methods.

Before discussing the historical implications of our results, we point out two general lessons that emerge from the analysis. The first is that AJ genetics defies simple demographic theories. Hypotheses such as a wholly Khazar, Turkish, or Middle-Eastern origin have been disqualified [4–7, 17, 55], but even a model of a single Middle-Eastern and European admixture event cannot account for all of our observations. The actual admixture history might have been highly complex, including multiple geographic sources and admixture events. Moreover, due to the genetic similarity and complex history of the European populations involved (particularly in Southern Europe [51]), the multiple paths of AJ migration across Europe [10], and the strong genetic drift experienced by AJ in the late Middle Ages [9, 16], there seems to be a limit on the resolution to which the AJ admixture history can be reconstructed.

The second lesson is the importance of evaluating the results of off-the-shelf tools using simulations when studying closely related populations. When simulating relatively old (≈1k years ago) Middle-Eastern and European admixture (particularly Southern European), we found many tools to be of limited utility (see, e.g., the section on *Alder*, *f*-statistics, and *TreeMix* and S1 Text sections 1 and 2 on *LAMP* and *PCAMask*). Further, while we eventually were able to extract useful information off *RFMix*’s local ancestries, the raw results were not very accurate: the accuracy per SNP was only ≈70%, the mean segment length was more than twice than expected, and the variance of the ancestry proportion per chromosome was overestimated. When jointly analyzing LAI and IBD sharing, the inferred proportion of IBD segments that were either not IBD or had a random ancestry assignment was as high as ≈35% ((1-*λ*) in Methods), although fortunately, we were able to account for that in our model. We note, though, that problems of this nature are not expected for recent admixture events between more diverged populations.

### Historical model and interpretation

Our model of the AJ admixture history is presented in Fig 7. Under our model, admixture in Europe first happened in Southern Europe, and was followed by a founder event and a minor admixture event (likely) in Eastern Europe. Admixture in Southern Europe possibly occurred in Italy, given the continued presence of Jews there and the proposed Italian source of the early Rhineland Ashkenazi communities [3]. What is perhaps surprising is the timing of the Southern European admixture to ≈24–49 generations ago, since Jews are known to have resided in Italy already since antiquity. This result would imply no gene flow between Jews and local Italian populations almost until the turn of the millennium, either due to endogamy, or because the group that eventually gave rise to contemporary Ashkenazi Jews did not reside in Southern Europe until that time. More detailed and/or alternative interpretations are left for future studies.

Recent admixture in Northern Europe (Western or Eastern) is consistent with the presence of Ashkenazi Jews in the Rhineland since the 10^th^ century and in Poland since the 13^th^ century. Evidence from the IBD analysis suggests that Eastern European admixture is more likely; however, the results are not decisive. An open question in AJ history is the source of migration to Poland in late Medieval times; various speculations have been proposed, including Western and Central Europe [2, 10]. The uncertainty on whether gene flow from Western Europeans did or did not occur leaves this question open.

### Caveats

The historical model we proposed is based on careful weighting of various methods and simulations, and we attempted to account for known confounders. However, it is possible that some remain. One concern is the effect of the narrow AJ bottleneck (effective size ≈300 around 30 generations ago [9, 16]) on local ancestry inference and on methods such as *TreeMix* and *f*-statistics. We did not explicitly model the AJ bottleneck in our simulations, though a bottleneck may have been artificially introduced since the number of independent haplotypes from each region used to generate the admixed genomes was very small. However, as we discuss in *Methods*, this is not expected to affect local ancestry inference, since each admixed chromosome was considered independently. Another general concern is that while we considered multiple methods, significant weight was given to the LAI approach; however, this may be justified as the LAI-based summary statistics were more thoroughly matched to simulations. Another caveat is that our estimation of the two-wave admixture model is based on heuristic arguments (the multiple European sources and the differential ancestry at IBD segments), and similarly for the admixture dates. The IBD analysis itself relies on a number of assumptions, most importantly that the error in LAI and in IBD detection is independent of the ancestry and that most of the moderately long IBD segments descend from a common ancestor living close to the time of the bottleneck (see S1 Text section 4 and S7 Fig).

A general concern when studying past admixture events is that the true ancestral populations are not represented in the reference panels. Here, while our AJ sample is extensive, our reference panels, assembled from publicly available datasets, are necessarily incomplete. Specifically, sampling is relatively sparse in North-Western and Central Europe (and particularly, Germany is missing), and sample sizes in Eastern Europe are small (10–20 individuals per population). In addition, we did not consider samples from the Caucasus (however, this is not expected to significantly affect the results [5]). We also neglected any sub-Saharan African ancestry, even though Southern European and Middle-Eastern populations (including Jews) are known to harbor low levels (≈5–10%) of such ancestry [49, 56]. Generally, bias will be introduced if the original source population has become extinct, has experienced strong genetic drift, or has absorbed migration since the time of admixture. Additionally, a reference population currently representing one geographic region might have migrated there recently. We note, however, that as we do not attempt to identify the precise identity of the ancestral source, but rather its very broad geographic region, some of the above mentioned concerns are not expected to significantly affect our results. Additionally, as we show in S1 Text section 3, our pipeline is reasonably robust to the case when the true source is absent from the reference panel. We note, though, that there may be other aspects of the real data that we are unaware of and did not model in our simulation framework that may introduce additional biases. Finally, we stress that our results are based on the working hypothesis that Ashkenazi Jews are the result of admixture between primarily Middle-Eastern and European ancestors, based on previous literature [4–8] and supported by the strong localization signal of the ME source to the Levant. Strong deviations from this assumption may lead to inaccuracies in our historical model.

### Future work

The admixture history of Ashkenazi Jews thus remains a challenging and partly open question. To make further progress, the natural next step is to use sequencing data. Whole-genomes are now available for several European populations (e.g., [57]) as well as for Ashkenazi Jews [9] and some Middle-Eastern groups [58]. The accuracy of LAI is expected to increase for sequencing data, as also noted for other analysis tools (e.g., [59]). Additionally, whole-genomes will make it possible to run analyses based on the joint allele frequency spectrum of AJ and other populations. In parallel, denser sampling of relevant European and Middle-Eastern populations (mostly from Central and Eastern Europe) will be required in order to refine the geographic source(s) of gene flow.

Beyond data acquisition, we identify three major methodological avenues for future research into AJ admixture. First, any improvement in the accuracy of local ancestry inference will translate into improved power to resolve admixture events. Second, methods will have to be developed for the inference of continuous and multi-wave admixture histories (e.g., [35]) under LAI uncertainty. At the same time, inference limits will have to be established for events temporally or geographically near, as we began to develop here (S1 Text section 6; see also [40]). Finally, one may use the signal in the lengths of IBD segments shared between AJ and other populations and within AJ to construct an admixture model (e.g., as in [60]), provided that we can reliably detect shorter segments than is currently possible.

## Methods

### Data collection

We merged the genotypes from all sources (Table 1, lifting over to hg19 whenever necessary), and removed cryptic relatives by detecting IBD segments (using *Germline* [44]) and removing one of each pair of individuals sharing more than 300cM. Individuals with a non-Ashkenazi genetic ancestry (defined to share less than 15cM, on average, with other AJ) were also removed. Other standard QC measures (carried out in *Plink* [61]) included removal of SNPs or individuals with a high no-call rate and eliminating SNPs with an ambiguous strand assignment. The genotypes of all individuals (of all ancestries) were jointly phased using *Shapeit* [62]. For the geographical localization analysis, we thinned the SNPs to eliminate LD using *Plink* (LD was measured in the entire dataset).

### Local ancestry inference using *RFMix*

*RFMix* was run using the TrioPhased option (see S1 Text section 1) and the generation parameter set to 30. Other parameters were set to default values. In each analysis involving the AJ individuals, we used a random subset of 200–500 individuals (out of overall 2540) in order to reduce running times. We did not use the expectation maximization (EM) option of *RFMix*, as simulations of ME/EU admixture demonstrated that inference accuracy was not improved by running the highly time-consuming EM step. Additionally, the EM step makes an iterative use of the admixed (Ashkenazi) genomes themselves in order to supplement the reference panels, thereby potentially introducing biases due to the excessive haplotype sharing in AJ. The final assigned local ancestries were the maximum-a-posteriori estimates. We verified that setting *RFMix*’s admixture time parameter to 50 generations did not change the inferred ancestry proportions.

#### Balancing the reference panels

To minimize biases in local ancestry inference, we ensured an equal number of European and Middle-Eastern individuals in the reference panel, as well as an equal number (30) of individuals from each European region (South, West, East, and Iberia). We used the same reference panel both for testing our simulations and for the AJ data, but the reference panel did not include the genomes used to create the simulated individuals (20 from each EU region and 20 from the Levant region). An initial inspection of our geographic localization pipeline demonstrated that Iberia had a much lower likelihood compared to the other regions (see also S1 Text section 3). We thus removed Iberia from our reference panel, which allowed us to significantly increase the number of individuals retained in the remaining regions (as Iberia had the smallest number of available genomes). Our final reference panel consisted of 273 EU and 273 ME individuals: 91 Eastern European, 91 Western European, 91 Southern European, 70 Druze, 77 Southern Middle-Eastern, and 126 Levantine individuals. We note that despite the smaller size of the reference panel when including Iberia, we were able to correctly identify the Iberian source in simulations of Iberia and Levant admixture. When studying the ME source, we used 50 individuals from each ME region (Levant, Druze, and Southern ME), as well as 50 from each of the three EU regions.

#### Global ancestry proportions

To infer the global ancestry proportions from *RFMix*, we simply used the proportion of SNPs classified as European/Middle-Eastern. We also attempted to infer global ancestry proportions using *ADMIXTURE* [63] (default parameters), either supervised or unsupervised, but we found in simulations that using *RFMix* outperformed *ADMIXTURE* (see also [34]).

### Simulations

For each admixed individual, we assumed that admixture (from all source populations) occurred at a single generation. The admixture parameters are the ancestry proportions contributed by each source and the admixture time *G* (generations ago). We generated haploid admixed chromosomes sequentially. The ancestry of each chromosomal segment was randomly selected, using the weight of each source (i.e., its ancestry proportions). We then randomly selected a chromosome from the chosen source population, and drew the segment length (in cM) from an exponential distribution with rate *G*/100. A haploid set of 22 chromosomes was then created for each individual. Diploid individuals were constructed by randomly pairing two sets of haploid chromosomes. Once generated, we did not evolve the simulated genomes.

### IBD sharing analysis

#### IBD segment detection

Five hundred random AJ individuals were selected for the IBD analysis. IBD segments were detected using *Germline* [44] with parameters *bits* = 64, *err_hom* = 1, *err_het* = 1, and a minimum length of 3cM. The detected segments were filtered with *Haploscore* [45] (cutoff 2) as well as by eliminating segments with more than 5% overlap with sequence gaps. In the analysis of the ancestry of the segments, we eliminated 0.25cM at each end of each segment to account for misidentification of their boundaries.

#### The ancestry of IBD segments

Denote by *p*_EU_ the genome-wide proportion of European ancestry in the AJ genomes, and assume it is known (e.g., 53%, as obtained from the LAI (*RFMix*) analysis). The goal of the IBD analysis is to compare *p*_EU_ to the proportion of EU ancestry in IBD segments. Complicating the analysis are (*i*) that the reported IBD segments are between diploid genomes, even though sharing is between single haplotypes; and (*ii*) errors in IBD detection and local ancestry inference. Nevertheless, the genome-wide expected effect of these confounders could be accounted for. To see this, denote by *λ* the proportion of genetic material in IBD segments that is both in true segments and whose inferred local ancestry is correct. The remaining genetic material (proportion 1-*λ*) is either not truly IBD or its inferred local ancestry is random. In both cases, the local ancestry assignment is EU with probability *p*_EU_ and ME with probability 1-*p*_EU_. Next, for each IBD segment, we determine the amount of genetic material (in cM) where the two individuals sharing the segment have given ancestries; specifically, at each SNP, each individual is either homozygous to EU ancestry, heterozygous, or homozygous to ME ancestry. Then, define the 3x3 observed IBD ancestry matrix ***A***_obs_, whose (*i*, *j*) entry corresponds to the proportion of genetic material in IBD segments where individual 1 has ancestry *i* and individual 2 has ancestry *j* (*i* and *j* can be homozygous-EU, heterozygous, or homozygous-ME). The matrix ***A***_rand_ is similarly defined, for regions that are either not truly IBD or have a random local ancestry assignment. ***A***_rand_ has expectation

$$A_{\text{rand}}=\left(\begin{array}{ccc} p_{\text{EU}}{}^{4} & 2p_{\text{EU}}{}^{3}\left(1-p_{\text{EU}}\right) & p_{\text{EU}}{}^{2}{\left(1-p_{\text{EU}}\right)}^{2}\\ 2p_{\text{EU}}{}^{3}\left(1-p_{\text{EU}}\right) & 4p_{\text{EU}}{}^{2}{\left(1-p_{\text{EU}}\right)}^{2} & 2p_{\text{EU}}{\left(1-p_{\text{EU}}\right)}^{3}\\ p_{\text{EU}}{}^{2}{\left(1-p_{\text{EU}}\right)}^{2} & 2p_{\text{EU}}{\left(1-p_{\text{EU}}\right)}^{3} & {\left(1-p_{\text{EU}}\right)}^{4} \end{array}\right).$$ (1)

(To simplify notation, and since there is no ambiguity, we use the same symbol for the matrix and its expectation.) For true IBD regions, we assume that all IBD segments descend from a common ancestor who lived around the time of the bottleneck (see below for justification). We denote the genome-wide EU ancestry at the onset of the bottleneck as *f*_EU_, which could be different than *p*_EU_: for example, a wave of post-bottleneck European gene flow would imply *f*_EU_ < *p*_EU_. At an IBD segment, the two individuals sharing it have, by definition, only three independent chromosomes (the one shared, and one other chromosome for each individual). The shared chromosome will be European with probability *f*_EU_, while the two other chromosomes will be European with probability *p*_EU_. Denote by ***A***_IBD_ the ancestry matrix at IBD segments with correct local ancestry assignment. The expectation of ***A***_IBD_ is

$$A_{\text{IBD}}=\left(\begin{array}{ccc} f_{\text{EU}}p_{\text{EU}}{}^{2} & f_{\text{EU}}p_{\text{EU}}\left(1-p_{\text{EU}}\right) & 0\\ f_{\text{EU}}p_{\text{EU}}\left(1-p_{\text{EU}}\right) & f_{\text{EU}}{\left(1-p_{\text{EU}}\right)}^{2}+\left(1-f_{\text{EU}}\right)p_{\text{EU}}{}^{2} & (1-f_{\text{EU}})p_{\text{EU}}\left(1-p_{\text{EU}}\right)\\ 0 & (1-f_{\text{EU}})p_{\text{EU}}\left(1-p_{\text{EU}}\right) & \left(1-f_{\text{EU}}\right){\left(1-p_{\text{EU}}\right)}^{2} \end{array}\right).$$ (2)

Note that no true IBD segments can have homozygous-EU ancestry for one individual and homozygous-ME ancestry for the other. Finally, we have

$$A_{\text{obs}}=\lambda A_{\text{IBD}}+\left(1-\lambda \right)A_{\text{rand}}.$$ (3)

We estimated the noise level *λ* by matching the (homozygous-EU/homozygous-ME) elements in ***A***_obs_ and ***A***_rand_, since these element are zero in ***A***_IBD_. Given *λ*, the empirical ***A***_IBD_ can be computed by rearranging Eq (3) (note that given *p*_EU_, ***A***_rand_ is known). We then estimated *f*_EU_ by minimizing the sum of absolute differences between the empirical and theoretical elements of ***A***_IBD_.

To determine the pre- and post-bottleneck admixture proportions, we assumed a model of pre-bottleneck admixture with proportions *f*_EU_:(1- *f*_EU_) and a post-bottleneck wave of European gene flow of magnitude *μ*_EU_. The total proportion of EU ancestry, *p*_EU_, can be written as *p*_EU_ = *μ*_EU_ + (1 - *μ*_EU_) ∙ *f*_EU_. Given the observed *p*_EU_ and the estimated *f*_EU_, *μ*_EU_ can be obtained.

In S1 Text section 4, we study the assumptions that IBD segments coalesce at the time of the bottleneck and that the IBD and LAI errors are independent of the ancestry of the segment.

### *Alder*, *f*_4_-statistics, and *TreeMix* analyses

We ran *Alder* [47] with default parameters (including an automatic detection of the minimal length cutoff), and with two reference populations. *f*_*4*_ statistics were computed using the implementation in the *TreeMix* package [50]. The *TreeMix* analysis itself was run with default parameters, except a block size (-k) of 500 (corresponding to ≈5MB, beyond the extent of typical LD).

### *GLOBETROTTER* analysis

On both simulations and real AJ data, *GLOBETROTTER* was run with default settings, as given in the example distributed with the program. For completeness, when generating only ancestry profiles (the proportion of ancestry contributed to the target population by each reference population), we set prop.ind = 1 and num.mixing.iterations = 0. When jointly inferring admixture events and proportions, we used, following the “Brahui-Yoruba” sample provided with *GLOBETROTTER*, boostrap.num = 20, props.cutoff = 0.001, and num.mixing.iterations = 5. In both modes, in the initial step of chromosome “painting” (*CHROMOPAINTER*), the AJ genomes were only allowed to be painted by donor/surrogate populations (Southern/Western/Eastern EU and Levant). To reduce the computational burden when running *GLOBETROTTER* on the real data, we used a random subset of 200 AJ individuals.

### Inferring admixture times using the distribution of ancestry proportions

A number of methods exist for the estimation of historical admixture times. Johnson et al. [18] fitted the number of ancestry switches; Pugach et al. [64] used a wavelet transform of the local ancestry along the genome; and Pool and Nielsen [65], as well as Gravel [35], fitted the distribution of segment lengths. However, these methods require an accurate identification of the boundaries of admixture segments, which is not always possible, especially for computationally phased data. Reich and colleagues [47–49] fitted the decay of admixture linkage disequilibrium (LD) with genetic distance (see main text), but their method can be confounded by background LD. Hellenthal et al. [21] recently proposed a promising approach based on the probability of two fixed loci to have given ancestries. Admixture parameters can also be inferred using more general demographic inference methods, e.g., based on the allele frequency spectrum [66, 67] or IBD sharing [60]; however, to use these methods one must specify and infer a model for the entire history. Recently, Rosenberg and colleagues [39, 68], Liang and Nielsen [69], and Gravel [35], derived analytical results for the moments of the ancestry proportion, namely the fraction of the chromosome that descends from each ancestral source. These ancestry proportions can be estimated (e.g., [63]) and matched to the theoretical moments for admixture time inference (e.g., [36, 37]). However, these methods do not make use of the information available in the entire distribution, and we therefore sought to derive it.

Our method assumes a simple admixture model, where the admixed population under investigation formed *t* generations ago as a result of merging of populations **A** and **B**, and where the proportion of ancestry contributed by **A** and **B** was *q* and 1 − *q*, respectively. We assume that lineages change along the chromosome due to recombination at rate *t* per Morgan. Ignoring genetic drift and constraints imposed by the underlying biparental pedigree (which is justified for admixture times around 10–100 generations ago and for typical human effective population sizes [40]), we assume that following recombination, the new source population is selected at random. Therefore, a recombination event will lead to a change of ancestry from **A** to **B** with probability 1 − *q* and from **B** to **A** with probability *q*. The lengths (in Morgans) of the chromosomal segments with **A** ancestry will therefore be exponentially distributed with rate (1 − *q*)*t*, and similarly for the **B** segments (rate *qt*) [35]. We neglect the first generation after admixture when **A** and **B** segments do not yet mix [35].

Given a chromosome of length *L* (Morgans), the ancestry along the chromosome can be modeled as a two-state process with states **A** and **B**, and with the distribution of segment lengths in each state given above. We are interested in the distribution of *x*, the fraction of the chromosome in state **A**. Adopting a result of Stam [38], the desired distribution is given by

$$\begin{array}{lll} f\left(x;L\right) & = & \left(1-q\right)e^{-qh}\delta \left(x\right)+qe^{-\left(1-q\right)h}\delta \left(1-x\right)\\ & + & q\left(1-q\right)he^{-h\left[\left(1-q\right)x+q\left(1-x\right)\right]}\left\{\left[qx+\left(1-q\right)\left(1-x\right)\right]\frac{I_{1}\left(2h\alpha \right)}{\alpha }+2I_{0}\left(2h\alpha \right)\right\}, \end{array}$$ (4)

Where *h* ≡ *tL*, $\alpha \equiv \sqrt{q\left(1-q\right)x\left(1-x\right)}$, and *I*_0_ and *I*_1_ are the modified Bessel functions of the first kind of order 0 and 1, respectively. Note the delta functions at *x* = 0 and *x* = 1, corresponding to the probability of the entire chromosome to have **B**-only or **A**-only ancestry, respectively. The mean ancestry proportion satisfies ⟨*x*⟩ = *q*, as expected. The variance is given by

$$\text{Var}\left[x\right]=\frac{2q\left(1-q\right)}{h^{2}}\left(e^{-h}+h-1\right)$$

in agreement with Eq (A16) in [35].

In practice, for unrelated individuals, phase switch errors are abundant, and hence it is difficult to accurately determine the ancestry proportion per chromosome. However, it is still possible to determine the diploid ancestry proportion, *y* = (*x*_1_ + *x*_2_)/2. Given that homologous chromosomes have independent histories (unless the population is extremely small [70]), its distribution, *f*_*d*_(*y*; *L*), can be computed from Eq (4) by convolution. Suppose we are now given the diploid ancestry proportions *y*_*ij*_ for individuals *i* = 1, …, *n* and chromosomes *j* = 1, …, 22 (where each chromosome has length *L*_*j*_). Assuming that chromosomes are independent both within and between individuals, the likelihood of the data is given by

$$\text{likelihood}=\prod_{i=1}^{n}\prod_{j=1}^{22}f\left(y_{ij};L_{j}\right)$$

We can then maximize the likelihood using a simple grid search over *q* and *t*. Simulation results with perfect knowledge of segment boundaries demonstrated that the method can correctly infer both *q* and *t* (S2 Fig), although the variance of the estimate increases with *t*.

We also considered a more complex historical model with an additional admixture event. Under this model, populations **A** and **B** had merged *t*_1_ generations ago, contributing proportions *q* and 1 − *q* to the admixed population. Then, *t*_2_ (< *t*_1_) generations ago, migrants from population **A** have replaced a fraction *μ* of the gene pool of the admixed population. No other events then take place until the present. Using the Markov process representation of the admixture process of Gravel [35], and using techniques of renewal theory, we were able to derive the distribution of the lengths of the **A** and **B** segments, which depend, in a complex way, on (*t*_1_, *t*_2_, *q*, *μ*). We then obtained an implicit expression for the distribution of the ancestry proportions. (More specifically, we obtained the Laplace transform of that distribution with respect to the chromosome length.) Mathematical details are given in S1 Text section 6. We observed that the distribution of ancestry proportions generated from the double admixture model can be fitted, for some parameter combination, with the pulse model (S1 Text section 6), and therefore, we did not use these theoretical results for inference.

Nonetheless, these results are useful for understanding the range of double admixture models that will be mapped into the same pulse admixture event. Specifically, under double admixture, the distribution of **B** segments lengths is exponential with rate *r* = *t*_1_ –(1 − *q*)(*t*_1_ –*μt*_2_), and the proportion of **B** ancestry is *M* = (1 − *q*)(1 − *μ*). Since for pulse admixture, *T* generations ago, *r* = (1 − *M*)*T*, the inferred time *T* under a pulse model satisfies

$$T\left(q+\mu -q\mu \right)=t_{1}-\left(1-q\right)\left(t_{1}-\mu t_{2}\right).$$ (5)

Given *T*, Eq (5) then imposes a constraint on the parameters of the model, in particular if *q* and *μ* can be independently estimated, as in our case.

## Supporting information

S1 Fig Principal Component Analysis (PCA) of the European and Middle-Eastern samples used as reference panels in our study. The analysis was performed using *SmartPCA* [25] with default parameters (except no outlier removal). The populations included within each region are listed in Table 1 of the main text. The PCA plot supports the partitioning of the European and Middle-Eastern populations into the broad regional groups used in the analysis.
(TIF)

S2 FigInference of admixture times using the distribution of ancestry proportions.We simulated an admixture pulse history under the Markovian Wright-Fisher model of [35]. The model assumes that the 2*N* haploid chromosomes in the current generation are formed by following a Markovian path along the 2*N* chromosomes of the previous generation, with ancestry changes occurring as a Poisson process with rate 1 per Morgan. Each chromosome in the first generation is assigned to population **A** or **B** with probabilities *q* and 1 − *q*, respectively, and the evolution of the chromosomes is traced for *t* generations. The model keeps record of the boundaries of the admixture segments along the generations, without explicitly simulating genotypes. We used *q* = 0.5, *L* = 2 Morgans, and *N* = 2500, and varied *t*. Ancestry proportions from pairs of chromosomes were averaged to simulate diploid individuals. We set the inferred *q* to the mean **A** ancestry, and used the distribution of ancestry proportions over the simulated individuals (Methods) to infer the admixture time *t*. Each dot in the plot shows the inferred time, $\hat{t}$, for one simulation. The dotted red line corresponds to $\hat{t}=t$, and the dashed purple line to the mean inferred time, $\langle \hat{t}\rangle$.(TIF)

S3 Fig The correlation between true and inferred ancestry proportions. We simulated 870 admixed individuals with 50% Southern European ancestry, 50% Levantine ancestry, and admixture time 30 generations ago. (A) Simulated vs *RFMix*-inferred Southern European ancestry proportion (*r*^2^ = 0.11). The regression line is plotted in blue. (B) The distributions of the simulated and *RFMix*-inferred ancestry proportions. The inferred proportions have a larger variance than the true ones, as well as a slightly lower mean (difference 0.03; for visualization, we shifted the *RFMix*-inferred distribution to match the true mean). A similar analysis with the EU component being entirely Western European resulted in a much higher correlation (*r*^2^ = 0.5), albeit with a larger bias (0.11 above than the true mean).
(TIF)

S4 Fig *f*_*4*_ statistics and potential tree topologies for the AJ history. The method is based on [48]. (A) Determining the most likely source of European gene flow into AJ. The statistic *f*_*4*_(X,YRI;AJ,ME) compares the amount of shared ancestry (solid black bar) between the paths connecting the European population X and Yoruba (green dashed lines) and the paths connecting AJ and Middle-Easterners (red dashed lines). The closer population X is to the true source of gene flow, the larger should be the *f*_*4*_ statistic. However, while we found higher values of *f*_*4*_ for Western and Eastern Europeans, simulations showed that this pattern is reproduced even under simulations with a predominantly Southern European source. (B) Estimating the European ancestry fraction. This is similar to (A), except that we computed the statistic *f*_*4*_(West-EU,YRI;South-EU,ME) (assuming that Southern Europe is the true source of European gene flow). As explained in Patterson et al. (Fig 2C therein), under the assumed tree topology, the ratio between the *f*_*4*_ statistics in (A) (with X = West-EU) and (B) should equal the fraction of European ancestry in AJ.
(TIF)

S5 Fig The effect of gene flow from the Middle-East into Southern EU on *f*_*4*_ statistics. Panels (A) and (B) demonstrate *f*_*4*_(West-EU,YRI;AJ,ME) and *f*_*4*_(South-EU,YRI;AJ,ME), respectively (cf S4A Fig). Paths from the Middle-East into AJ are indicated with red arrows; paths from YRI to Western or Southern Europe with green arrows. The *f*_*4*_ statistic is proportional to the total overlap between these paths (black bars). Whereas panel (B) (*f*_*4*_(South-EU,YRI;AJ,ME)) has more overlapping branches than in (A), migration from the Middle-East into Southern EU introduces a branch where the arrows run in opposite directions (patterned bar). Hence, the observed *f*_*4*_ statistic in (B) may be lower (depending on branch lengths) than in (A), even if Southern EU is the true source of gene flow into AJ.
(TIF)

S6 Fig The graph structure of the AJ/EU/ME population histories, as estimated by *TreeMix* [50]. (A) Real data. (B) Simulated AJ data (along with the actual EU and ME genomes used in our study). Two hundred genomes were simulated according to a 4-way model with 50% Middle-East, 35% South-EU, 12% East-EU, and 3% West-EU ancestries, with the mixing occurring 30 generations ago. The arrows indicate gene flow.
(TIF)

S7 Fig IBD segment accuracy vs the proportion of the IBD segment with Middle-Eastern (ME) ancestry. The proportion was averaged over all four haplotypes involved in each IBD segment (i.e., the two haplotypes of each of the two individuals sharing the segment). IBD accuracy was measured using *Haploscore*, which is proportional to the number of genotyping and phasing errors required for the segment to be truly IBD (i.e., lower scores are better). In a linear regression analysis of the *Haploscore* vs the segment length and the ME ancestry, the coefficient of the ME ancestry was <0.01.
(TIF)

S1 Text Supplementary results and methods. Section 1: local ancestry inference (LAI). Section 2: PCAMask. Section 3: Robustness of the LAI-based inferred ancestry proportions. Section 4: The IBD sharing analysis. Section 5: The *GLOBETROTTER* analysis. Section 6: Mathematical details on the distribution of ancestry proportions under a two-wave admixture model.
(PDF)
