# Supplementary figures and images for: The time and place of European admixture in Ashkenazi Jewish history
Source: PLoS Genet. 2017 Apr 4;13(4):e1006644. doi: 10.1371/journal.pgen.1006644 (PMC5380316; doi:10.1371/journal.pgen.1006644)

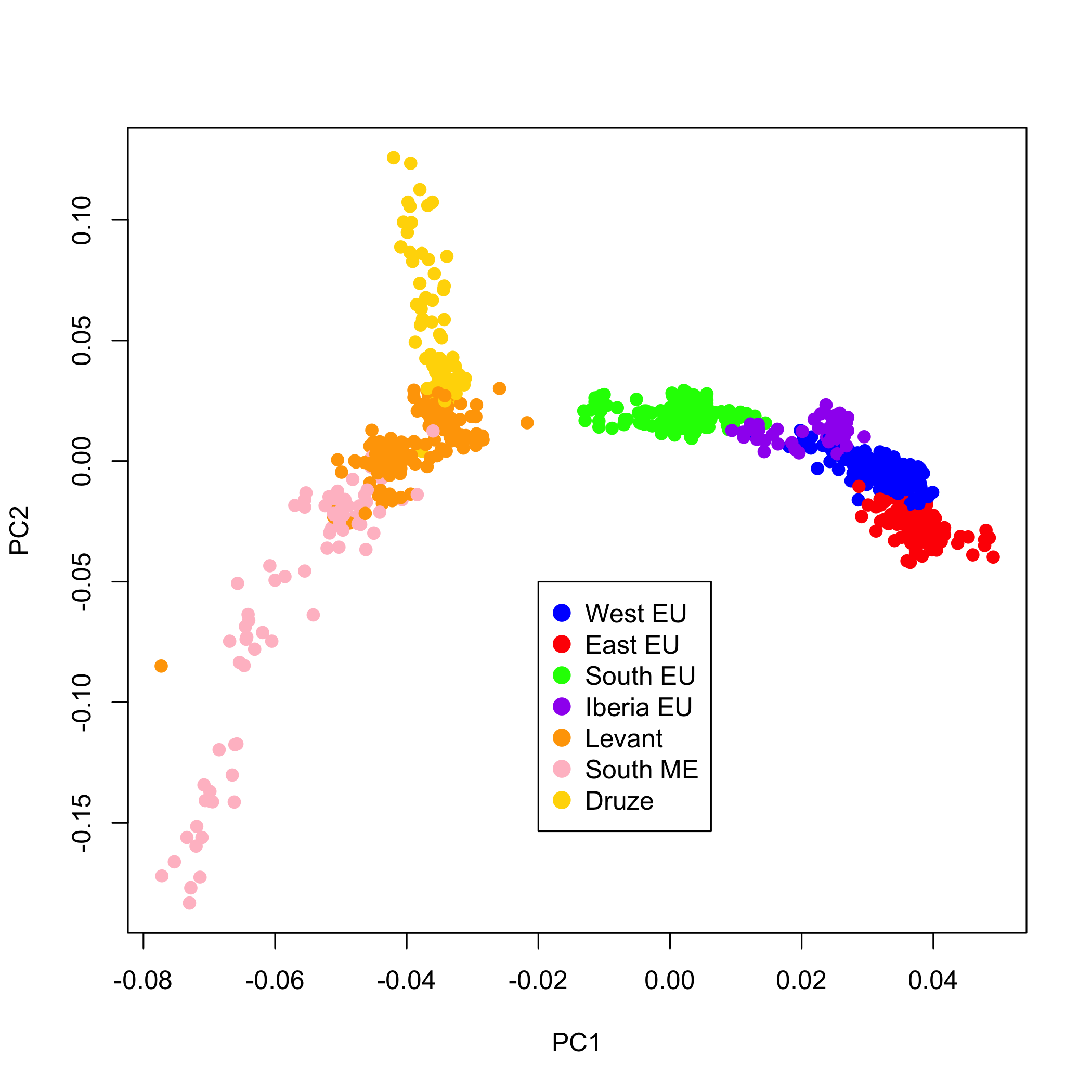

Supplement: S1 Fig — The analysis was performed using SmartPCA [25] with default parameters (except no outlier removal). The populations included within each region are listed in Table 1 of the main text. The PCA plot supports the partitioning of the European and Middle-Eastern populations into the broad regional groups used in the analysis. (TIF) [file pgen.1006644.s001.tif]

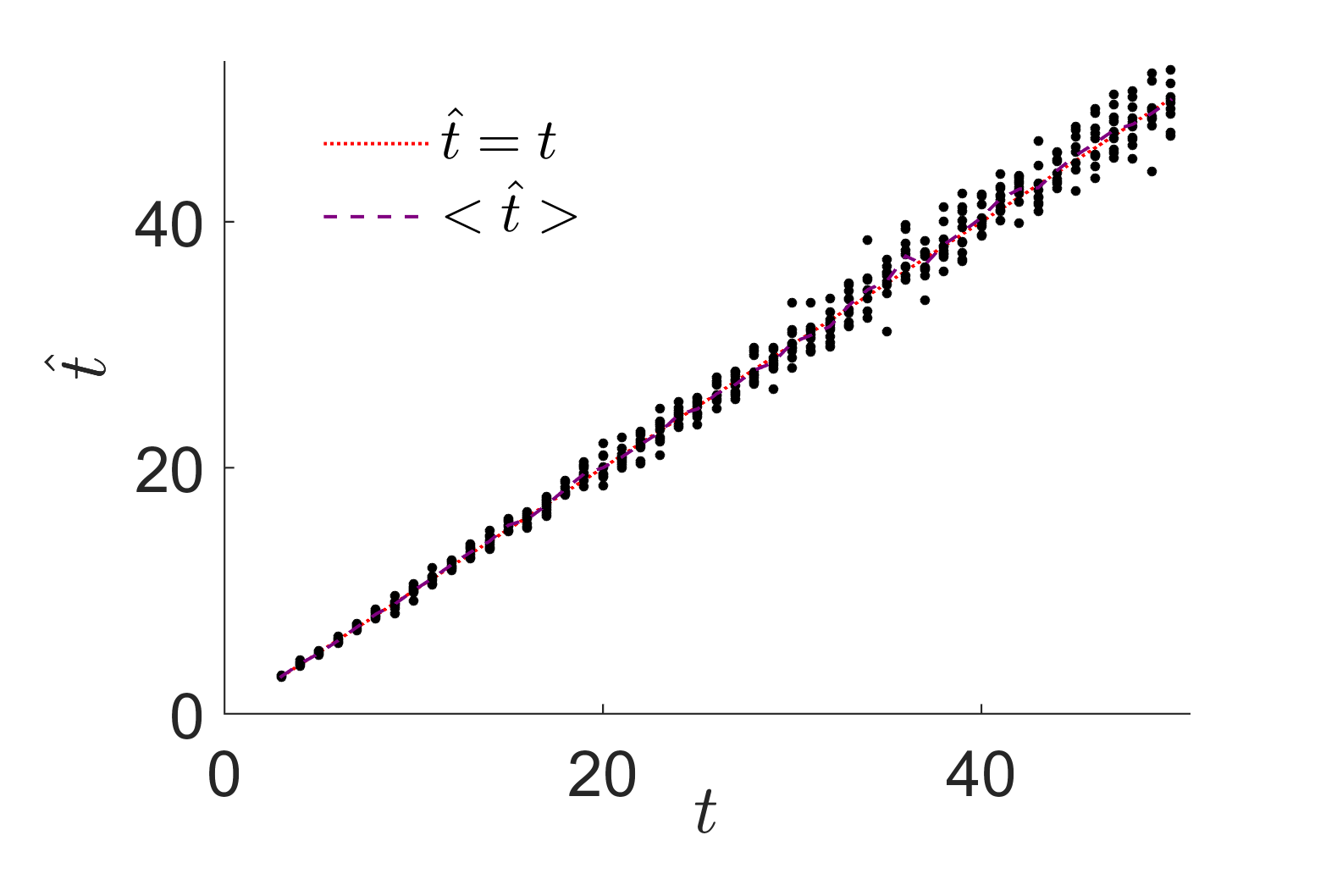

Supplement: S2 Fig — We simulated an admixture pulse history under the Markovian Wright-Fisher model of [35]. The model assumes that the 2N haploid chromosomes in the current generation are formed by following a Markovian path along the 2N chromosomes of the previous generation, with ancestry changes occurring as a Poisson process with rate 1 per Morgan. Each chromosome in the first generation is assigned to population A or B with probabilities q and 1 − q, respectively, and the evolution of the chromosomes is traced for t generations. The model keeps record of the boundaries of the admixture segments along the generations, without explicitly simulating genotypes. We used q = 0.5, L = 2 Morgans, and N = 2500, and varied t. Ancestry proportions from pairs of chromosomes were averaged to simulate diploid individuals. We set the inferred q to the mean A ancestry, and used the distribution of ancestry proportions over the simulated individuals (Methods) to infer the admixture time t. Each dot in the plot shows the inferred time, t^, for one simulation. The dotted red line corresponds to t^=t, and the dashed purple line to the mean inferred time, 〈t^〉. (TIF) [file pgen.1006644.s002.tif]

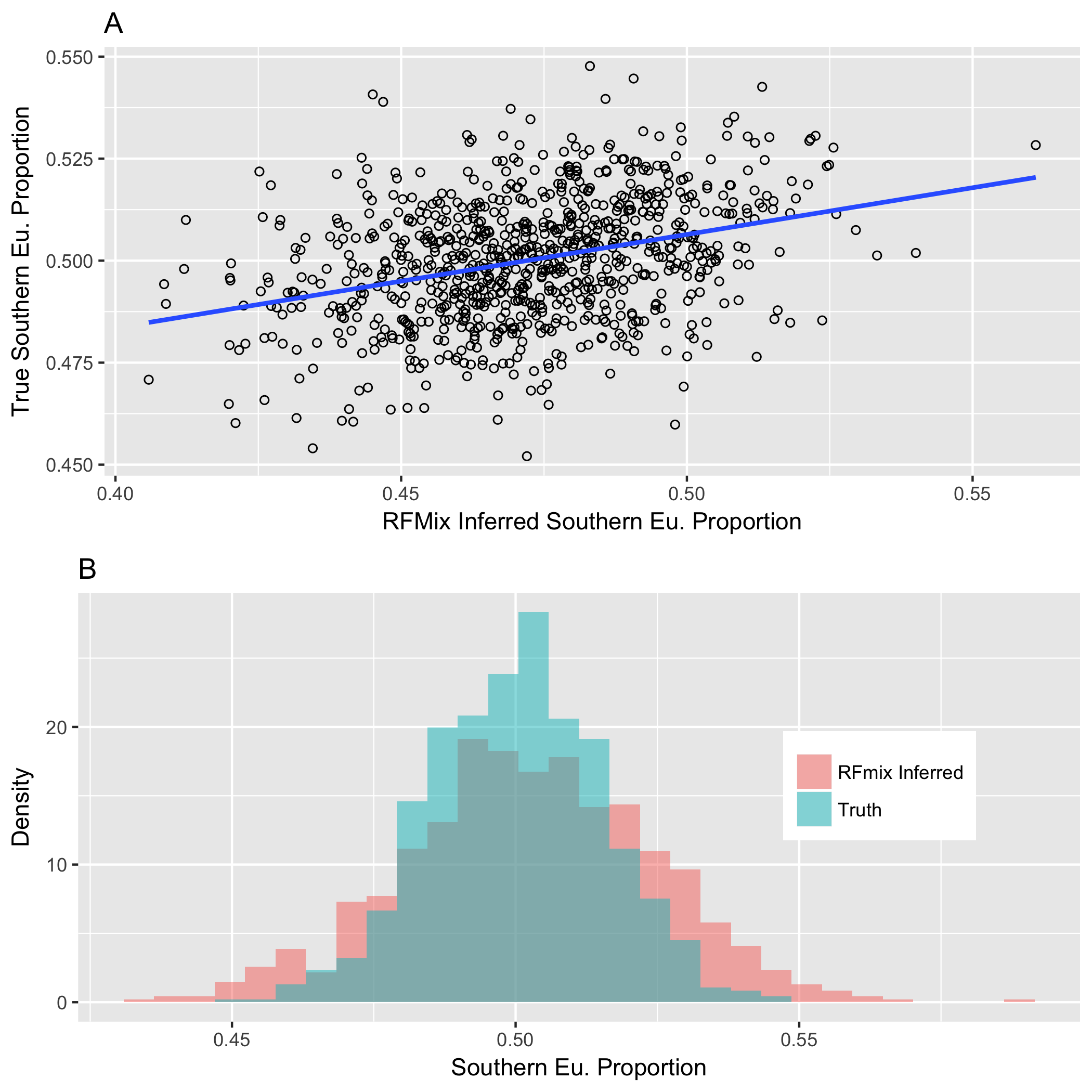

Supplement: S3 Fig — We simulated 870 admixed individuals with 50% Southern European ancestry, 50% Levantine ancestry, and admixture time 30 generations ago. (A) Simulated vs RFMix-inferred Southern European ancestry proportion (r2 = 0.11). The regression line is plotted in blue. (B) The distributions of the simulated and RFMix-inferred ancestry proportions. The inferred proportions have a larger variance than the true ones, as well as a slightly lower mean (difference 0.03; for visualization, we shifted the RFMix-inferred distribution to match the true mean). A similar analysis with the EU component being entirely Western European resulted in a much higher correlation (r2 = 0.5), albeit with a larger bias (0.11 above than the true mean). (TIF) [file pgen.1006644.s003.tif]

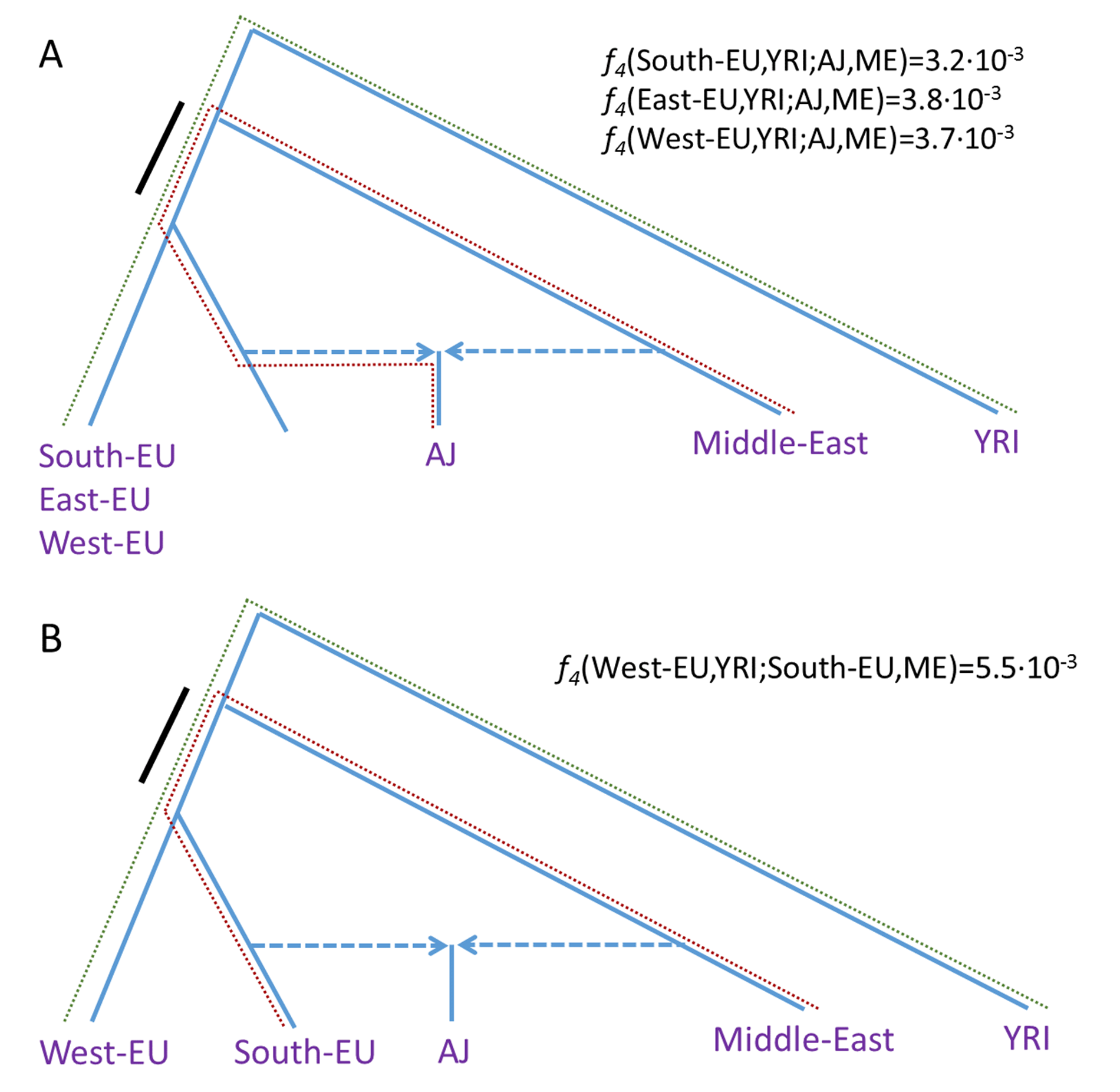

Supplement: S4 Fig — The method is based on [48]. (A) Determining the most likely source of European gene flow into AJ. The statistic f4(X,YRI;AJ,ME) compares the amount of shared ancestry (solid black bar) between the paths connecting the European population X and Yoruba (green dashed lines) and the paths connecting AJ and Middle-Easterners (red dashed lines). The closer population X is to the true source of gene flow, the larger should be the f4 statistic. However, while we found higher values of f4 for Western and Eastern Europeans, simulations showed that this pattern is reproduced even under simulations with a predominantly Southern European source. (B) Estimating the European ancestry fraction. This is similar to (A), except that we computed the statistic f4(West-EU,YRI;South-EU,ME) (assuming that Southern Europe is the true source of European gene flow). As explained in Patterson et al. (Fig 2C therein), under the assumed tree topology, the ratio between the f4 statistics in (A) (with X = West-EU) and (B) should equal the fraction of European ancestry in AJ. (TIF) [file pgen.1006644.s004.tif]

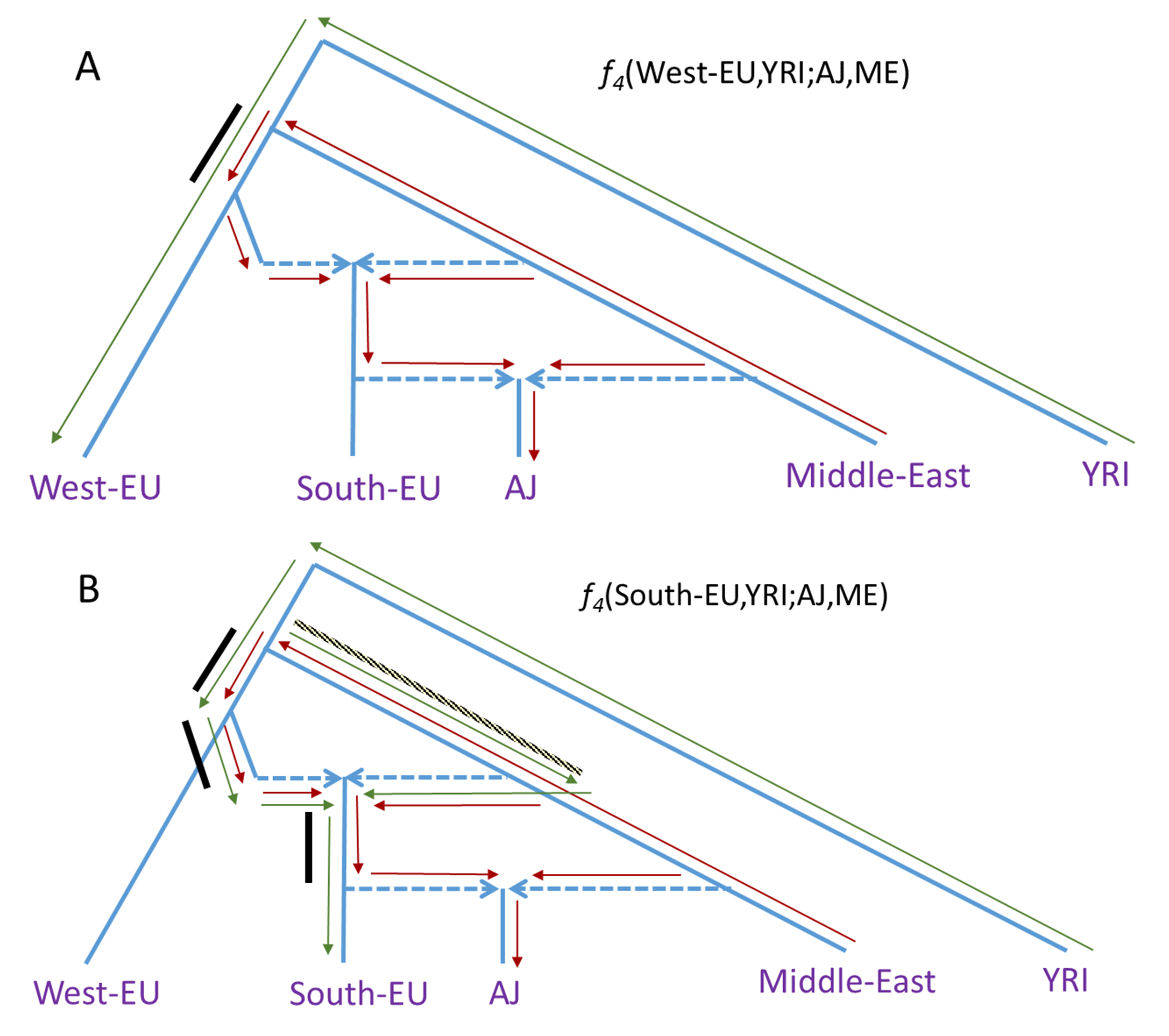

Supplement: S5 Fig — Panels (A) and (B) demonstrate f4(West-EU,YRI;AJ,ME) and f4(South-EU,YRI;AJ,ME), respectively (cf S4A Fig). Paths from the Middle-East into AJ are indicated with red arrows; paths from YRI to Western or Southern Europe with green arrows. The f4 statistic is proportional to the total overlap between these paths (black bars). Whereas panel (B) (f4(South-EU,YRI;AJ,ME)) has more overlapping branches than in (A), migration from the Middle-East into Southern EU introduces a branch where the arrows run in opposite directions (patterned bar). Hence, the observed f4 statistic in (B) may be lower (depending on branch lengths) than in (A), even if Southern EU is the true source of gene flow into AJ. (TIF) [file pgen.1006644.s005.tif]

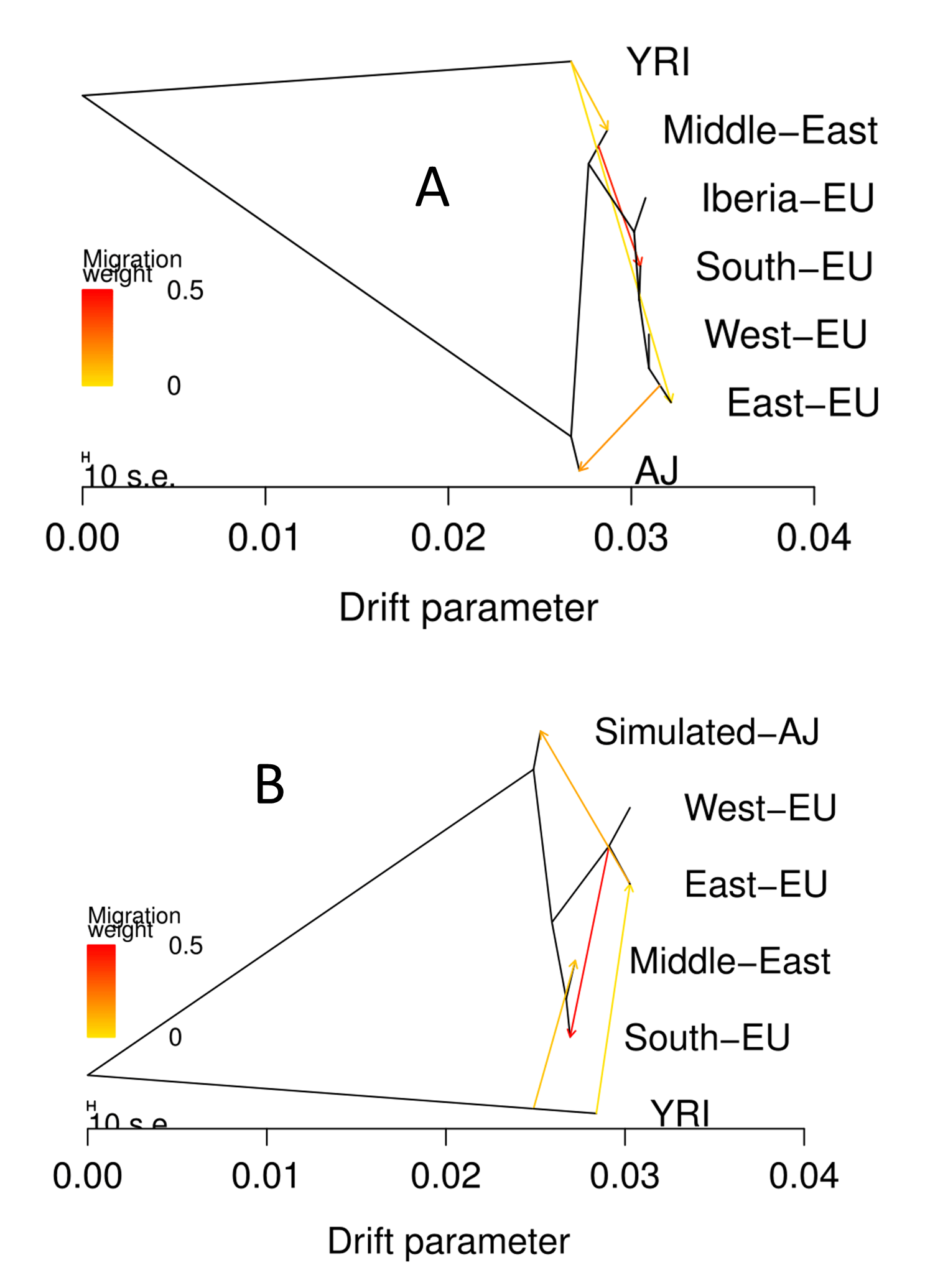

Supplement: S6 Fig — (A) Real data. (B) Simulated AJ data (along with the actual EU and ME genomes used in our study). Two hundred genomes were simulated according to a 4-way model with 50% Middle-East, 35% South-EU, 12% East-EU, and 3% West-EU ancestries, with the mixing occurring 30 generations ago. The arrows indicate gene flow. (TIF) [file pgen.1006644.s006.tif]

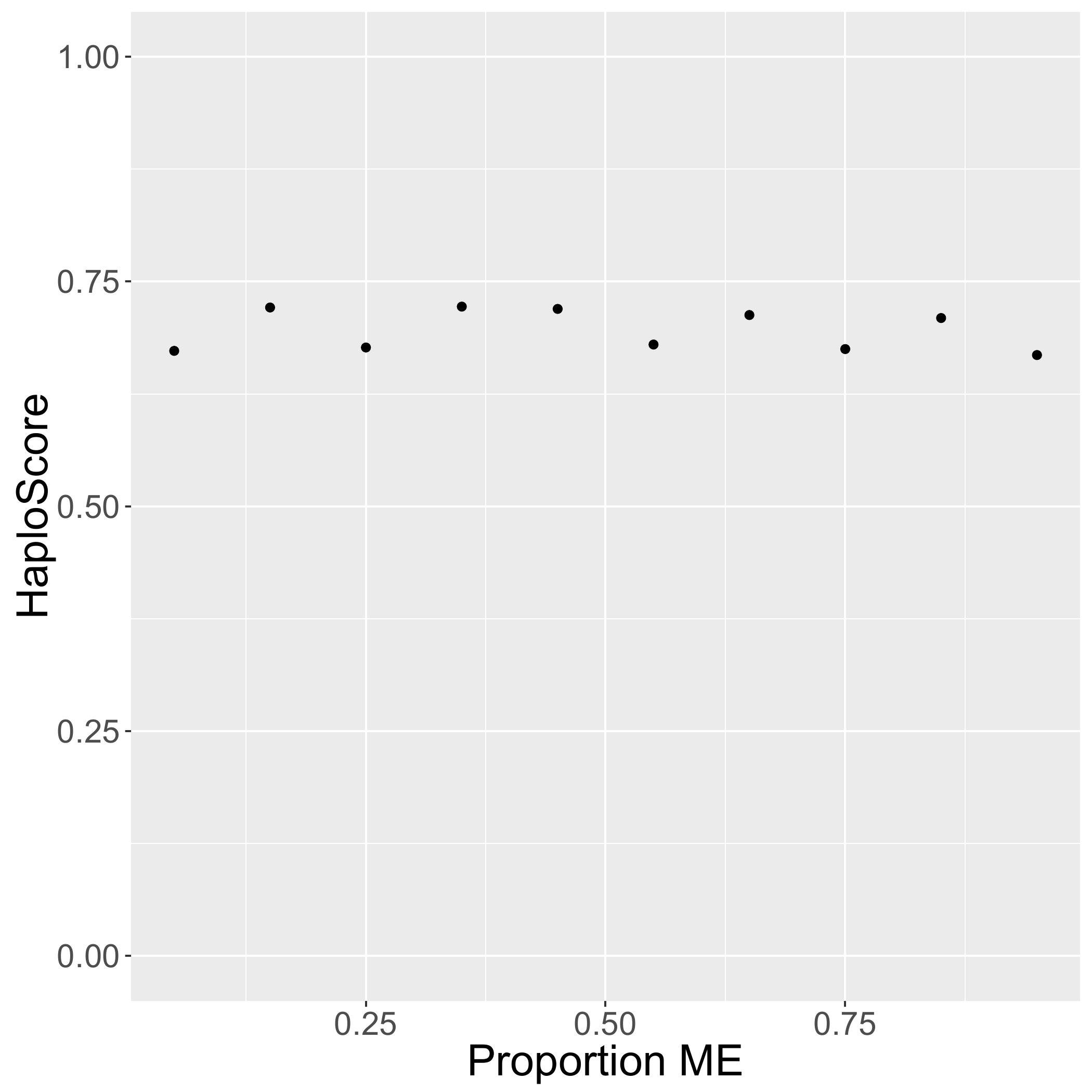

Supplement: S7 Fig — The proportion was averaged over all four haplotypes involved in each IBD segment (i.e., the two haplotypes of each of the two individuals sharing the segment). IBD accuracy was measured using Haploscore, which is proportional to the number of genotyping and phasing errors required for the segment to be truly IBD (i.e., lower scores are better). In a linear regression analysis of the Haploscore vs the segment length and the ME ancestry, the coefficient of the ME ancestry was <0.01. (TIF) [file pgen.1006644.s007.tif]
