# Supplementary material for: The time and place of European admixture in Ashkenazi Jewish history
Source: PLoS Genet. 2017 Apr 4;13(4):e1006644. doi: 10.1371/journal.pgen.1006644 (PMC5380316; doi:10.1371/journal.pgen.1006644)
Supplement: S1 Text — Section 1: local ancestry inference (LAI). Section 2: PCAMask. Section 3: Robustness of the LAI-based inferred ancestry proportions. Section 4: The IBD sharing analysis. Section 5: The GLOBETROTTER analysis. Section 6: Mathematical details on the distribution of ancestry proportions under a two-wave admixture model. (PDF) [file pgen.1006644.s008.pdf]

S1 Text

Section 1: Local ancestry inference

Testing local ancestry inference (LAI) tools

LAI tools are known to be highly effective in distinguishing ancestries at a continental level (e.g., African vs European ancestry); however, at the subcontinental level LAI may be noisy. Thus, before selecting an LAI tool, we used simulated admixed genomes from pairs of populations from the 1000 Genomes Project (phase I [1]) to determine the accuracy (as reflected by the proportion of sites whose ancestry was correctly classified) of *LAMP-LD* [2] and *RFMix* [3]. Both programs use a window-based framework; *LAMP-LD* uses a generative approach using Hidden Markov Models, whereas *RFMix* employs a discriminative modeling approach using random forests. For our initial tests, we found that while *LAMP-LD* was effective for distantly related populations (e.g., admixture between YRI and CEU), it had a much lower resolution for closer populations (e.g., TSI and FIN, which are populations with F_{ST} around 1%, about the same as that between AJ and EU/ME populations). In contrast, *RFMix* was more effective at distinguishing TSI/FIN ancestries, and subsequent analyses demonstrated its ability to distinguish (albeit with noise) also Middle-Eastern and European ancestries.

Robustness to phasing errors

While our local ancestry inference pipeline ran on perfectly phased data in our simulations, the AJ genotypes were computationally phased. To determine whether phase switch errors are a concern, we performed the following experiment. We simulated 100 individuals with admixture occurring 30 generations ago and with ancestry proportions 50% Southern European and 50% Levantine. After pairing sets of simulated chromosome, we randomly scrambled the phase, and then ran *Shapeit* to computationally re-phase all genotypes. We then re-ran the simulated genomes through our entire pipeline to infer the most likely geographic source. We found that the results essentially remained the same as when working with perfect phase, namely the genomes were localized to the true underlying European and Middle-Eastern subcontinental ancestry (Southern Europe and Levant) and the number of sites correctly classified as EU/ME did not change. Since computationally phasing each set of simulated genomes would have been extremely computationally expensive, the originally simulated haplotypes were used in all analyses.

The effect of filtering low-quality SNPs

We initially filtered SNPs according to *RFMix*'s posterior probabilities (a measure of the confidence of the SNP to come from a specific ancestry), as we observed in simulations that filtering led to higher accuracy of LAI. However, we found that filtering led to biases in our geographic localization pipeline. Specifically, in simulations, we were able to correctly localize a Southern European source only when we did not filter any SNPs. We attribute this result to the Middle-Eastern gene flow into Southern European (specifically, Italian) populations (e.g., [4]) and our use of a diverse reference panel that includes multiple European ancestries. These factors are expected to result in lower confidence in classifying Southern EU segments compared to segments from other European sources. In turn, filtering low quality SNPs would lead to

disproportionately retaining segments of Northern European origin, thus wrongly localizing the EU segments even if the true source is Southern Europe. To guarantee the unbiased nature of our pipeline, we therefore did not filter any SNPs in all subsequent analyses.

Section 2: *PCAMask*

PCAMask is a software tool that performs principal component analysis restricted to the SNPs in each individual that derive from a specific ancestry [5, 6]. In theory, such a tool should be able to pinpoint the subcontinental ancestries of admixed individuals, but the utility of *PCAMask* on admixture between closely related populations was unknown. Running *PCAMask* on the AJ genomes (along with the reference panels described in the main text), we found that occasionally, the European component of the AJ genomes clustered around Southern Europe and that the Middle-Eastern component of the AJ genomes clustered around the Levant region, in concordance with the results presented in the main text. Nevertheless, we did not include these results due to a number of technical issues (see also [7]). Specifically, we found that in certain situations, the algorithm did not reach convergence and some AJ individuals were localized far away from the main AJ cluster. In addition, we found that the program did not appear to control for the number of admixed individuals: we noticed that increasing the number of AJ individuals led to their inconsistent placement. Finally, we compared the clustering of the reference EU and ME individuals between *PCAMask* and the commonly used *SmartPCA* tool [8], and noticed discrepancies in the clustering pattern. We therefore leave a more rigorous interpretation of *PCAMask*'s results to future work.

Section 3: Robustness of the LAI-based inferred ancestry proportions

Confidence intervals for the inferred ancestry proportions

To obtain confidence intervals for the ancestry proportions of each EU region inferred in Figure 2 of the main text, we resampled AJ individuals 1000 times with replacement, and used linear regression in the region near the real AJ data point to obtain the simulated values matching each bootstrap iteration. We used a similar procedure for the admixture time estimated in Figure 3 of the main text. We stress that this procedure accounts only for the sampling noise, but the magnitude of other biases is clearly higher. For example, note the difference in the Southern EU ancestry proportion between panels (A) and (B) of Figure 2 (31% vs 37%, respectively), as well as the results from *GLOBETROTTER*.

The proportion of Levant ancestry

When generating Figure 2 of the main text, we fixed the Levant ancestry to 50% and varied the amount of the different European ancestries. To determine whether our results are sensitive to the assumed proportion of Levant ancestry, we fixed the proportion of Western and Eastern European ancestry to 8% each, and varied the proportion of Southern European ancestry between 20% and 80% in increments of 5%, with the remaining ancestry being Levantine. The best match to the AJ data (in terms of the proportion of chromosomes classified as Southern European) was obtained for Levant ancestry proportions of 49% (leaving Southern EU with 35%). In another experiment, we fixed the Levantine ancestry to 60% and varied the Southern EU ancestry in increments of 5% (the remaining ancestry being Eastern European). The best

match to the AJ data was found at 30% Southern European ancestry, close to the 34% inferred using the original pipeline.

The contribution of Iberia

We removed Iberia from our reference panel at an early stage of the analysis. To directly estimate the Iberia ancestry proportions in AJ, we simulated admixed genomes with Levant ancestry proportions of 50%, Southern EU proportions of 34%, and varying Western/Eastern EU and Iberia ancestry between 0 and 16%. The closest match to the real AJ data (in terms of the proportion of chromosomes classified as Iberia) was obtained when the Iberia component was 2%.

Exclusion of the true ancestral source from the Middle-Eastern or European reference panels

Here, we study the robustness of our results to not having available samples from the precise ancestral source of AJ. This is the case if the original source population has gone extinct, experienced strong genetic drift, absorbed migration since the time of admixture, or otherwise was missed from our sample. To investigate the expected effect on our results, we study the case when the true source used for simulating the admixed genomes is removed from the reference panel.

For the case of the Middle-East, we arbitrarily selected Jordanians, and simulated admixed genomes with 50% Jordanian ancestry, along with 34% Southern EU ancestry, 8% Western EU ancestry, and 8% Eastern EU ancestry (the ancestry proportions inferred in the main text, Figure 2). We then excluded Jordanians from the ME reference panel, and attempted to reconstruct the ancestry proportions from each European source (assuming the total EU ancestry was 50%), following precisely the pipeline described in Figure 2 for the real AJ data. Our estimate for the Southern European ancestry proportion was 32.5%, very close to the true simulated proportion (34%). We thus conclude that our inference pipeline is reasonably robust to exclusion of the precise ancestral ME source from the panel.

For the case of Europe, we focused on Western EU, since our sampling there was relatively sparse (and specifically, did not include Germany). We simulated admixed genomes with 50% French and 50% Levant ancestry, and then removed French from the Western EU panel. The proportion of chromosomes whose European component was correctly classified as Western EU was 49%, compared to 33% classified as Southern EU. (In comparison, under a simulation of 50% Southern EU and 50% Levant ancestries, the proportion of chromosomes classified as Southern EU was 53% compared 29% Western EU.) Thus, even in the absence of the specific ancestral source from the Western European reference panel, we were still able to correctly infer its regional affiliation.

The simulated admixture time

In the simulations used in Figure 2 of the main text, we assumed that the admixture time was 30 generations ago, which is the value we estimated both here and previously [9]. To determine the robustness of our results to deviations from this estimate, we repeated the inference procedure, but when setting the simulated admixture time to 20 generations. We also set the admixture time parameter of *RFMix* to 50 generations. We fixed the Levantine ancestry to 50% and increased the Southern EU ancestry fraction in increments of 2% (the remaining ancestry was 8% Western EU and 8% Eastern EU). We found

that the best fit to the AJ data was obtained at Southern EU ancestry of 38%, close to the originally inferred proportions of 34%.

Section 4: The IBD sharing analysis

The coalescence time of an IBD segment

The IBD sharing analysis described in the main text assumed that given that a site is in an IBD segment, it coalesces around the time of the bottleneck. The exact posterior distribution of the coalescence time of a segment of length (in Morgans) between m_1 and m_2 is given by (e.g., [10, 11])

$$(1)\quad g(t) = \frac{h(t)e^{-\int_0^t h(\tau)d\tau}\left[(1+4N_A m_1 t)e^{-4N_A m_1 t} - (1+4N_A m_2 t)e^{-4N_A m_2 t}\right]}{\int_0^\infty h(t')e^{-\int_0^{t'} h(\tau)d\tau}\left[(1+4N_A m_1 t')e^{-4N_A m_1 t'} - (1+4N_A m_2 t')e^{-4N_A m_2 t'}\right]dt'},$$

where N_A is the ancestral population size (in diploids), $h(t)$ is the coalescence probability per generation (more precisely, the inverse of the population size when scaled by $2N_A$), and the time t is scaled by $2N_A$. For a bottleneck that has reduced the population size from 10k to 300 individuals 30 generations ago and was followed by a rapid expansion to 1M individuals, as inferred for AJ [9, 11], we find that coalescence times of segments of length [3,7]cM are narrowly distributed, with ≈86% of events taking place within [20,40] generations ago. This suggests that the ancestry of IBD segments reflects predominantly the ancestry during the generations close to the bottleneck. Information on deviations from this assumption is encoded in the lengths of the segments and may be modeled in future work.

The dependence of the errors on the ancestry

The IBD sharing analysis relies on the assumption that false positive IBD and/or uninformed LAI are independent of the ancestry. To determine whether IBD detection accuracy varies across ancestries, we plotted, in S7 Figure, the *Haploscore* for each segment against its ME ancestry, averaged over the four haplotypes involved, and observed that the *Haploscore* was nearly constant across the entire range of ME ancestries. To investigate how the LAI error varies across ancestries, we simulated admixed genomes with ancestry proportions 50:34:8:8% Levantine, Southern EU, Eastern EU, and Western EU, respectively. We then ran *RFMix* and determined the proportion of SNPs correctly classified as either ME or EU. The Levant SNPs were classified correctly 70.5% of the time, compared to 68.8% for the EU SNPs, which supports using an ancestry-independent error rate. A subtle caveat is, though, the relatively low classification accuracy of Southern EU SNPs: 62.8%, compared to 80.1% and 82.3% for Eastern and Western EU, respectively. To model the effect of this result on our estimate of the pre-bottleneck ancestry proportions, and assuming that pre-bottleneck gene flow was mostly Southern European (Figure 5 of the main text), denote the true pre-bottleneck ME ancestry as f_{ME}, the estimated pre-bottleneck ME ancestry as g_{ME} (58%, see *Results* in the main text), the probability of correctly classifying a ME SNP as p(ME→ME) (70.5%), and the probability of incorrectly classifying a Southern EU SNP as ME as p(SEU→ME) (37.2%). The following equation then approximately holds: $g_{ME}=f_{ME}\cdot p(ME\rightarrow ME)+(1-f_{ME})\cdot p(SEU\rightarrow ME)$. Solving for f_{ME}, we obtain f_{ME}=62%, higher than the uncorrected estimate of 58%. Thus, our approach is conservative, in the sense that if biased, it has likely underestimated the evidence for an elevated pre-bottleneck ME ancestry, and consequently, the evidence for post-bottleneck European gene flow.

Section 5: *GLOBETROTTER*

Comparing EU ancestry proportion estimates between *RFMix* and *GLOBETROTTER*

The estimate of the total EU ancestry from the *RFMix* analysis was 53%, consistently with our previous estimate of ≈50-55% based on whole-genome data [9], the estimate from the f_4 analysis (when corrected by simulations), and the experiment mentioned in Supplementary Text S3 above ("The proportion of Levant ancestry"). In contrast, the estimate from *GLOBETROTTER* [12] was 70%, among which 55% was Southern European. We find that reconciling these estimates is difficult, as evidence exists to support both the LAI-based estimate and the *GLOBETROTTER* based estimate.

To test *GLOBETROTTER*, we simulated individuals with ancestry proportions 8% Western EU, 8% Eastern EU, 34% Southern EU, and 50% Levant, with admixture happening 30 generations ago. *GLOBETROTTER* was able to recover all proportions within ±1% of the simulated ones. For simulations with ancestry proportions 70% Southern EU and 30% Levant, the *GLOBETROTTER*-inferred EU proportions were slightly overestimated at 73%. Thus, given *GLOBETROTTER*'s estimated 70% EU ancestry in AJ, the implied true EU ancestry in AJ would be 67%. On the other hand, *RFMix*'s inferred proportions were underestimated at 62%. However, the bias for simulated 50% Southern EU and 50% Levant ancestries was much lower, with *RFMix*'s inferred EU proportions at 48%.

In conclusion, there remains some uncertainty regarding the amount of EU ancestry in AJ, to be further investigated in future studies. It seems plausible that the true EU ancestry proportions are midway between *RFMix*'s and *GLOBETROTTER*'s estimates. For most of this paper we assumed the *RFMix* estimate (≈55%), as (*i*), it is supported by other lines of evidence; and (*ii*) the results from the two modes of *GLOBETROTTER* were discordant (see below).

GLOBETROTTER-inferred admixture parameters on simulated data

We used simulations to test the ability of *GLOBETROTTER* to jointly infer admixture time and sources [12]. The simulated individuals had 70% Southern EU and 30% Levant ancestries, with admixture occurring 30 generations ago. *GLOBETROTTER* inferred two sources: the first, comprising of 39% of the total ancestry, was a mixture of 15% Southern European ancestry and 85% Levant ancestry; the second source was 1% Eastern European, 28% Western European, and 71% Southern European. Thus, the true Southern EU ancestry proportions were not properly recovered (inferred 49% vs simulated 70%), although the global EU ancestry was correctly inferred (67% vs simulated 70%). The inferred admixture time was overestimated at 40 generations.

The number of admixture events

GLOBETROTTER is able to infer multiple admixture events, although for AJ, the inferred history included only a single event. This might be at odds with our hypothesis (supported by the IBD analysis) of pre-bottleneck admixture with Southern Europeans followed by post-bottleneck admixture with (possibly) Eastern Europeans. However, we note that one source of ancestral population inferred by GLOBETROTTER is a mixture of Southern EU and Levant, which may correspond to the earlier event we have identified. It

is also possible that the two events are too close to be teased apart, or that the inference of the admixture times is confounded by the severe AJ bottleneck [12].

Bibliography

1. Genomes Project, C., et al., *An integrated map of genetic variation from 1,092 human genomes.* Nature, 2012. **491**(7422): p. 56-65.

2. Baran, Y., et al., *Fast and accurate inference of local ancestry in Latino populations.* Bioinformatics, 2012. **28**(10): p. 1359-67.

3. Maples, B.K., et al., *RFMix: a discriminative modeling approach for rapid and robust local-ancestry inference.* Am J Hum Genet, 2013. **93**(2): p. 278-88.

4. Pardo-Seco, J., et al., *A genome-wide study of modern-day Tuscans: revisiting Herodotus's theory on the origin of the Etruscans.* PLoS One, 2014. **9**(9): p. e105920.

5. Moreno-Estrada, A., et al., *Reconstructing the population genetic history of the Caribbean.* PLoS Genet, 2013. **9**(11): p. e1003925.

6. Johnson, N.A., et al., *Ancestral components of admixed genomes in a Mexican cohort.* PLoS Genet, 2011. **7**(12): p. e1002410.

7. Browning, S.R., et al., *Local Ancestry Inference in a large US-Based Hispanic/Latino Study: Hispanic Community Health Study / Study of Latinos (HCHS/SOL).* G3 (Bethesda), 2016.

8. Patterson, N., A.L. Price, and D. Reich, *Population structure and eigenanalysis.* PLoS Genet, 2006. **2**(12): p. e190.

9. Carmi, S., et al., *Sequencing an Ashkenazi reference panel supports population-targeted personal genomics and illuminates Jewish and European origins.* Nat. Commun., 2014. **5**: p. 4835.

10. Carmi, S., et al., *A renewal theory approach to IBD sharing.* Theor. Popul. Biol., 2014. **97**: p. 35-48.

11. Palamara, P.F., et al., *Length distributions of identity by descent reveal fine-scale demographic history.* Am J Hum Genet, 2012. **91**(5): p. 809-22.

12. Hellenthal, G., et al., *A genetic atlas of human admixture history.* Science, 2014. **343**(6172): p. 747-51.

S1 Text section 6: The distribution of ancestry proportions under two-wave admixture

1 The distribution of ancestry proportions under general distributions of segment lengths

In the main text, we considered a simple admixture pulse model, under which the distribution of segment lengths in **A** and **B** is exponential with rates $(1-q)t$ and qt, respectively. Under that model, the distribution of ancestry proportions was available in a closed form. Under a more complex admixture history, we assume that the distribution of the length of **A** and **B** segments take the general form $g_{\mathbf{A}}(\ell)$ and $g_{\mathbf{B}}(\ell)$. We still assume that **A** and **B** segments are independent (see below). The process can then be modeled as a two-state process. We start on the left end of the chromosome in state **A** or **B** with probabilities $p_A = \langle \ell_A \rangle \,/\, (\langle \ell_A \rangle + \langle \ell_B \rangle)$ and $1 - p_A$, respectively (where $\langle \ell_A \rangle$ and $\langle \ell_B \rangle$ are the mean segment lengths), and draw a random segment length from the selected ancestry. When the first segment terminates, we switch ancestries and draw a segment length from the other ancestry, and so on until we reach the end of the chromosome.

The distribution of x, the **A** ancestry proportion, can be computed in Laplace space by extending renewal theory methods developed in the physics domain (e.g., [1, 2]). Let s be the Laplace pair of L (the total chromosome length) and u the Laplace pair of $L_A = xL$ (the total chromosome length covered by A segments). We then transform the density $f(L_A; L)$ (from which the density of x can be easily obtained) to $\hat{f}(u; s)$. After some calculations using renewal theory, we eventually obtain,

$$\hat{f}(u;s) = \frac{s\left[1 - \hat{g}_{\mathbf{A}}(s+u)\hat{g}_{\mathbf{B}}(s)\right] + u\left[1 - \hat{g}_{\mathbf{B}}(s)\right]\left\{1 - p_A\left[1 - \hat{g}_{\mathbf{A}}(s+u)\right]\right\}}{s(s+u)\left[1 - \hat{g}_{\mathbf{A}}(s+u)\hat{g}_{\mathbf{B}}(s)\right]}. \quad (1)$$

In the above equation, $\hat{g}_{\mathbf{A}}(s)$ and $\hat{g}_{\mathbf{B}}(s)$ are the Laplace transforms ($\ell \to s$) of $g_{\mathbf{A}}(\ell)$ and $g_{\mathbf{B}}(\ell)$. The details of the derivation are somewhat tedious and are therefore omitted. It can be shown, using Eq. (1), that the mean ancestry proportion $\langle x \rangle$ approaches p_A as $L \to \infty$. It can be also shown that Eq. (1) reduces to Eq. (5) in the main text for the admixture pulse model.

2 Conditions under which consecutive segments are independent

To study complex admixture histories, we use the model developed by Gravel [3] (section *General incoming migration in the absence of drift* and Figure 3 therein). Gravel proposed that the ancestry along the chromosome could be described by a Markov process, whose states correspond to the identity of the source population (i.e., **A** or **B**), combined with the time when each segment entered the admixed population. Gravel then derived the transition rates for any general admixture history. While the extended state space process is Markovian under any history, consecutive **A** and **B** segment lengths are no longer independent. However, further examination demonstrates that as long as migration beyond the initial event is limited to just one population, consecutive segment lengths remain independent.

3 A two-wave admixture model

Consider a model where populations **A** and **B** have merged t_1 generations ago, contributing proportions q and $1-q$ to the admixed population. Then, t_2 ($< t_1$) generations ago, migrants from population **A** have replaced a proportion μ of the gene pool of the admixed population. No other events then take place until the present. The corresponding Markov process, using Gravel's method [3], has three states: A_1, A_2, and B, representing migrant segments from **A** at time t_1, from **A** at time t_2, and from **B** (at time t_1), respectively. Let us compute the distributions of the lengths of **A** and **B** segments.

The transition rate is t_1 when at states A_1 and B, and t_2 when at A_2. It can be shown that once a transition is made, the next state is chosen according to the following transition probability matrix

$$\mathbf{P} = \begin{pmatrix} q\left(1-\mu\frac{t_2}{t_1}\right) & \mu\frac{t_2}{t_1} & (1-q)\left(1-\mu\frac{t_2}{t_1}\right) \\ q(1-\mu) & \mu & (1-q)(1-\mu) \\ q\left(1-\mu\frac{t_2}{t_1}\right) & \mu\frac{t_2}{t_1} & (1-q)\left(1-\mu\frac{t_2}{t_1}\right) \end{pmatrix}. \tag{2}$$

The states are ordered as (A_1, A_2, B) and $\mathbf{P}_{ij}$ $(i,j = 1,2,3)$ is the probability to jump from state i to state j. Note that we neglected the first generation after admixture, during which **A** and **B** segments do not yet mix [3].

It is now easy to see that **B** segment lengths are distributed exponentially with rate $t_1(1-\mathbf{P}_{B,B})$, or

$$\begin{aligned} g_{\mathbf{B}}(\ell) &= t_1\left[1-(1-q)\left(1-\mu\frac{t_2}{t_1}\right)\right]\exp\left\{-t_1\ell\left[1-(1-q)\left(1-\mu\frac{t_2}{t_1}\right)\right]\right\} \\ &= \left[t_1-(1-q)(t_1-\mu t_2)\right]\exp\left\{-\ell\left[t_1-(1-q)(t_1-\mu t_2)\right]\right\}. \end{aligned} \tag{3}$$

This equation was also (implicitly) derived in [4] in a different way. For the **A** segments, define $g_{A_1}(\ell)$ as the distribution of **A** segment lengths, *when the*

process entered the $\boldsymbol{A}$ *states at state* A_1, and similarly for $g_{A_2}(\ell)$. Since the process enters A_1 and A_2 from B (with the possible exception at the leftmost end of the chromosome), the distribution of $\mathbf{A}$ segments satisfies

$$g_{\mathbf{A}}(\ell) = \frac{\mathbf{P}_{B,A_1}}{1-\mathbf{P}_{B,B}} g_{A_1}(\ell) + \frac{\mathbf{P}_{B,A_2}}{1-\mathbf{P}_{B,B}} g_{A_2}(x). \tag{4}$$

To find $g_{A_1}(\ell)$ and $g_{A_2}(\ell)$, we can write integral equations,

$$\begin{aligned} g_{A_1}(\ell) &= \mathbf{P}_{A_1,B} t_1 e^{-t_1\ell} + \int_0^\ell t_1 e^{-t_1 y} \left[\mathbf{P}_{A_1,A_1} g_{A_1}(\ell-y)dy + \mathbf{P}_{A_1,A_2} g_{A_2}(\ell-y)\right] dy \\ g_{A_2}(\ell) &= \mathbf{P}_{A_2,B} t_2 e^{-t_2\ell} + \int_0^\ell t_2 e^{-t_2 y} \left[\mathbf{P}_{A_2,A_1} g_{A_1}(\ell-y)dy + \mathbf{P}_{A_2,A_2} g_{A_2}(\ell-y)\right] dy. \end{aligned} \tag{5}$$

We solved these equations by Laplace transform ($\ell \to s$). Using the convolution theorem,

$$\begin{aligned} \hat{g}_{A_1}(s) &= \frac{t_1}{t_1+s}\left[\mathbf{P}_{A_1,B} + \mathbf{P}_{A_1,A_1}\hat{g}_{A_1}(s) + \mathbf{P}_{A_1,A_2}\hat{g}_{A_2}(s)\right] \\ \hat{g}_{A_2}(s) &= \frac{t_2}{t_2+s}\left[\mathbf{P}_{A_2,B} + \mathbf{P}_{A_2,A_1}\hat{g}_{A_1}(s) + \mathbf{P}_{A_2,A_2}\hat{g}_{A_2}(s)\right]. \end{aligned} \tag{6}$$

These are two linear equations in two variables ($\hat{g}_{A,1}(s)$ and $\hat{g}_{A,2}(s)$), which are easily solved. Then, $g_{A,1}(\ell)$ and $g_{A,2}(\ell)$ are obtained by Laplace transform inversion. We then use Eq. (4) to obtain $g_{\mathbf{A}}(\ell)$. We carried out these steps in MATHEMATICA, leading to the final result,

$$g_{\mathbf{A}}(\ell) = \frac{(1-q)e^{-\gamma\ell/2}\left[C_1\sinh(\beta\ell/2) + C_2\cosh(\beta\ell/2)\right]}{\beta\left[qt_1 + \mu t_2(1-q)\right]} \tag{7}$$

where $\gamma = t_2 + (1-q)(t_1 - t_2\mu)$, $\beta = \sqrt{\gamma^2 - 4t_1t_2(1-q)(1-\mu)}$,

$$C_1 = q^2(t_1-\mu t_2)^3 - q(t_1-\mu t_2)\left[t_1^2 - t_1t_2 - 2t_2^2\mu(1-\mu)\right] + t_2^2\mu(1-\mu)\left[t_1 - t_2(1-\mu)\right],$$

and

$$C_2 = \left[q(t_1-\mu t_2)^2 + \mu(1-\mu)t_2^2\right]\beta.$$

Now that we have $g_{\mathbf{A}}$ and $g_{\mathbf{B}}$ (Eqs. (7) and (3), respectively), we can use Eq. (1) for the distribution of the ancestry proportions. We inverted $\hat{f}(u;s)$ with respect to u using MATHEMATICA and then numerically with respect to s to obtain $f(x;L)$.

4 Simulation results and fitting

We ran simulations of the Markovian Wright-Fisher model described by Gravel [3]. The model assumes $2N$ haploid individuals (chromosomes). Each chromosome in the current generation is formed as a mixture of the chromosomes of the

previous generation. Ancestry changes occur as a Poisson process with rate 1 (per Morgan), and at each ancestry change, the ancestral chromosome is chosen randomly out of all $2N$ available chromosomes. In the pulse admixture model, each chromosome in the first generation is assigned to population **A** or **B** with probabilities q and $1-q$, respectively, and the evolution of the chromosomes is traced for t generations. The two-wave model is the same (with overall time t_1), except that at t_2 generations ago, each chromosome is replaced by a whole-**A** chromosome with probability μ.

Representative simulation results are shown in Supplementary Text Figure 1. It can be seen that our theory matches the empirical data very well. However, the empirical distribution can also be fitted well by a distribution corresponding to an admixture pulse model, with parameter q_{pulse} close to the expected mean $(\mu + q(1-\mu))$ and t_{pulse} intermediate between t_1 and t_2. This suggests that inference based on the more complex model may not have sufficient evidence, under some conditions, to justify the additional admixture event.

References

[1] C. Godrèche and J. M. Luck. Statistics of the occupation time of renewal processes. *J. Stat. Phys.*, 104:489, 2001.

[2] G. Margolin and E. Barkai. Aging correlation functions for blinking nanocrystals, and other on-off stochastic processes. *J. Chem. Phys.*, 121:1566–1577, 2004.

[3] S. Gravel. Population genetics models of local ancestry. *Genetics*, 191:607–619, 2012.

[4] X. Ni, X. Yang, W. Guo, K. Yuan, Y. Zhou, Z. Ma, and S. Xu. Length distribution of ancestral tracks under a general admixture model and its applications in population history inference. *Sci. Rep.*, 6:20048, 2016.

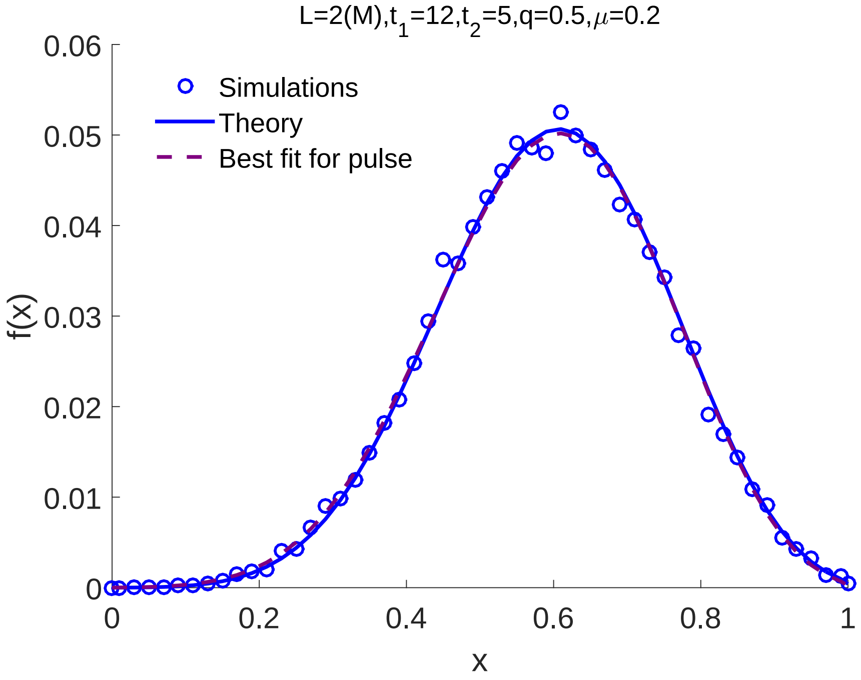

Supplementary Text Figure 1: Two-wave admixture: simulation and theory. We simulated a two-wave admixture model according to a Markovian Wright-Fisher model [3] with $N = 2500$. The other model parameters are indicated in the title of the figure. We recorded the fraction of each chromosome that descends from the **A** population, and plotted the histogram of the ancestry proportions (circles). The theory that we developed (Eqs. (1), (3), and (7)) is plotted as a solid (blue) line. We then fitted a pulse admixture model with just two parameters (q and t), by matching the mean and variance of the empirical data. The distribution of the ancestry proportions under the pulse model (Eq. (4) in the main text) is plotted as a dashed (purple) line. The best fit for t was 9.7, intermediate between t_1 and t_2.
